# Interleukin-27-adipose-derived mesenchymal stromal cell-based gene therapy attenuates inflammation in lipopolysaccharide-induced acute respiratory distress syndrome

**DOI:** 10.1186/s13287-025-04647-1

**Published:** 2025-09-29

**Authors:** Grace E. Mulia, Viviana Galindo, Ying-Cheng Chen, Janelle W. Salameh, Marxa L. Figueiredo

**Affiliations:** 1 Department of Basic Medical Sciences, College of Veterinary Medicine, Purdue University, 625 Harrison St, LYNN 2177, West Lafayette, IN 47904 USA; 2 Purdue Interdisciplinary Life Sciences (PULSe), Purdue University, West Lafayette, IN USA; 3 Weldon School of Biomedical Engineering, Purdue University, West Lafayette, IN USA

**Keywords:** Interleukin-27, Adipose-derived mesenchymal cells, Mesenchymal stromal cells, Acute respiratory distress syndrome

## Abstract

**Background:**

Acute respiratory distress syndrome (ARDS) is a lung inflammatory condition associated with the accumulation of fluid edema and cell infiltrates into the alveolar space along with dysregulation of the immune response. Current therapeutics are limited to palliative care, i.e., mechanical ventilators, thus highlighting the need to develop targeted therapeutic for ARDS. Interleukin-27 (IL-27) is a multifunctional cytokine with the capability for immune modulation. Our interest lies in exploring the properties of IL-27, particularly as an anti-inflammatory cytokine that functions as an antagonist of IL-6 signaling, as an inducer of anti-viral genes, as a promoter of tissue repair, and as a regulator of both the innate and adaptive immune responses, possessing promising potential as a therapeutic for ARDS.

**Methods:**

To overcome the challenge of repeated administration due to the short half-life of cytokines, we utilized a cell-based gene therapy approach. An IL-27-expressing plasmid was transfected into adipose mesenchymal stromal cells (ASC) that serve as the gene therapy carriers. For in vitro studies, we treated mono- and co-culture lung lipopolysaccharide (LPS)-induced lung epithelial and monocytes/macrophages cell line with IL-27-expressing ASC (IL-27 ASC) conditioned media (CM) to determine the effects on pro-inflammatory gene expression. For in vivo studies, male C57BL/6 mice were intratracheally injected with LPS (5 mg/kg) and treated either PBS, ASC, or IL-27 ASC (5 × 10^5^ cells/mouse) 24 h after LPS instillation. Measurements of gene expression, histopathological changes, cell infiltrations, and protein content in the bronchoalveolar lavage fluid and RNA-seq analysis were performed for the in vivo studies.

**Results:**

IL-27 ASC CM reduced pro-inflammatory gene expression of lung epithelial and macrophages cultured in both mono- and co-culture systems. Additionally, IL-27 ASC were able to reduce pro-inflammatory markers, decrease cell infiltration into the lungs, promote genes and immune cells involved in tissue repair, and rebalance innate and adaptive immunity in an LPS-induced in vivo model.

**Conclusions:**

Collectively, our in vitro and in vivo results show promising potential for IL-27 cell-based gene therapy as a treatment for ARDS.

**Supplementary Information:**

The online version contains supplementary material available at 10.1186/s13287-025-04647-1.

## Background

Acute respiratory distress syndrome (ARDS) is a form of lung inflammation associated with pulmonary edema and characterized by the presence of diffuse pulmonary infiltrates and lung stiffness [1]. ARDS can be caused by direct lung injury, including trauma, bacterial, viral, or fungal infection, as well as indirect lung injury, such as sepsis [2]. An observational study on ARDS encompassing 29,000 + intensive care unit patients found that ~ 10.4% suffered from ARDS, of which 30% were mild, 46.6% were moderate, and 23.4% were severe [2]. The mortality rate in severe cases of ARDS remains high, with 40% of severe cases resulting in death. Currently, there is no cure for ARDS, and patients are only given supportive care, i.e., mechanical ventilation [2], to alleviate their symptoms. This highlights the dire need for targeted therapeutics to treat ARDS.

Interleukin-27 (IL-27) is a pleiotropic, multifunctional cytokine composed of two subunits, Epstein Barr virus-induced gene 3 protein (EBI3) and IL27p28 [3, 4]. IL-27 signals through the heterodimeric IL-27 receptor (IL-27R), consisting of IL-27Rα and gp130 [4]. The receptor is expressed in a variety of cell types, including immune cells (T cells, B cells, NK cells, macrophages, dendritic cells), epithelial cells, endothelial cells, and stem cells [4, 5]. IL-27 is typically expressed by antigen-presenting cells (APC) and activates multiple immune signaling pathways. IL-27 has been shown to have anti-inflammatory functions, acting as an antagonist of IL-6 [6], and signals to counter IL-1 and TNF-α pathways [7]. Additionally, IL-27 is capable of promoting tissue repair [8] and rebalancing innate and adaptive immunity [9]. To date, no studies have investigated the potential of IL-27 in reducing inflammation in the ARDS context, making it a critical area for further exploration.

However, utilizing IL-27 as a therapeutic cytokine presents some challenges. Due to the short half-life of cytokines, repeated administration of recombinant IL-27 would be necessary for an effective therapeutic regimen. To address this limitation, we designed a cell-based targeted gene delivery approach using an IL-27 expressing plasmid. We chose a non-viral episomal approach because it raises fewer biosafety concerns, is more cost-effective, highly reproducible, and has a low risk of insertional mutagenesis that is important for a gene therapy with transient expression, as inflammation eventually resolves. Additionally, we utilized adipose-derived mesenchymal stromal cells (ASC) as our gene delivery carrier. Mesenchymal stromal cells (MSC) have long been shown to have anti-inflammatory properties and several studies - both pre-clinical and clinical - have been conducted to investigate their efficacy in alleviating ARDS [10–12]. Compared to MSC derived from bone marrow, umbilical cord, or blood, ASC are more favorable due to their less invasive acquisition method, while retaining similar efficacy to MSC from other sources [13]. Taken together, we hypothesized that human ASC expressing IL-27 can reduce inflammation in models of ARDS.

To test this hypothesis, we electroporated human ASC with an IL-27 expressing plasmid and evaluated its efficacy in both in vitro and in vivo models of bacterially induced ARDS using lipopolysaccharide (LPS). The LPS-induced model was chosen as it is one of the most widely studied models of ARDS and mimics ARDS caused by pneumonia, which is one of the most common causes of ARDS in the clinical setting [2]. Results from this study serve as the first step to further our understanding of the potential use of IL-27 and ASC as effective therapeutics to attenuate ARDS. Therefore, the findings of this study are specific to LPS-induced ARDS and should not be generalized to other etiologies such as viral or sterile injury. Future studies exploring the efficacy of our therapeutic in additional in vivo models of ARDS are warranted to improve clinical relevance.

## Methods

### Cell culture

Human epithelial carcinoma cell line A549, from the American Type Culture Collection (ATCC, Manassas, VA), was cultured in Dulbecco’s modified Eagle’s Medium (DMEM) (Corning, Corning, NY) supplemented with 10% Fetal Bovine Serum (FBS) (Hyclone, Cytiva, Marlborough, MA) and 1% antibiotic-antimycotic (Anti-Anti) (Gibco, Waltham, MA). The normal human epithelial cell line BEAS-2B, from ATCC, was cultured in pre-coated plates and maintained in Bronchial Epithelial Cell Growth Medium (BEGM) with all the recommended supplements from the manufacturer (Lonza, Morristown, NJ). The pre-coat mixture for BEAS-2B contained 10 µg/mL fibronectin from human plasma (Sigma-Aldrich, St. Louis, MO), 30 µg/mL collagen (Cultrex, Minneapolis, MN), and 10 µg/mL bovine serum albumin (Jackson ImmunoResearch Labs, West Grove) dissolved in BEGM medium [14]. Plates containing the pre-coat mixture were incubated at 37 °C with 5% CO_2_ for at least 2 h before the mixture was aspirated and the plates were used for cell culture. The monocytic cell line THP-1, kindly provided by Dr. Herman Sintim, was cultured in Roswell Park Memorial Institute (RPMI) 1640 medium (Corning), supplemented with 10% heat-inactivated FBS (ATCC).

Human adipose mesenchymal stromal cells (hASC) from a male Caucasian donor (age 53, body mass index 30.02) were obtained through Obatala Sciences (New Orleans, LA). The hASC demonstrated key features of mesenchymal stromal cells (MSC), including confirmed adipogenic and osteogenic differentiation, supporting a multipotent potential. Flow cytometry analysis at passage 0 indicated expression of key MSC surface markers, alongside low-level expression of hematopoietic markers, consistent with a heterogeneous early-passage MSC population (Suppl. Fig S1). Importantly, the cells passed sterility testing and exhibited metabolic activity (12.7% alamarBlue reduction) and a doubling time of 2.1 days, supporting their suitability for research applications where early-passage heterogeneity is acceptable and potentially advantageous for modeling diverse biological responses.

Human ASC were cultured on fibronectin-coated plates in modified medium (ASC medium). ASC medium contained 60% DMEM (Corning), 40% MCDB-201 medium (Sigma-Aldrich), 5% FBS (Hyclone), 1x Insulin-transferrin-selenium (Corning), 1 nM dexamethasone (Thermo Fisher, Waltham, MA), 10 ng/mL epidermal growth factor (Gibco), 0.1 µM ascorbic acid (Thermo Fisher), and 1% Anti-Anti, following previously outlined protocols [15].

### Vectors

Two different sets of vectors were used for in vitro and in vivo studies due to differences in species-specific IL-27 (human vs. mouse) [16]. Hence, a human IL-27 expressing plasmid was used for the in vitro studies while a mouse IL-27 expressing plasmid was used for in vivo studies. Backbones were chosen based on prior research from our group showing optimal expression from these vectors for mouse or human IL-27 [17].

The first pair used for in vitro studies consisted of pNLF1-secNluc (Promega, Madison, WI) and Pep7.1 (human IL-27 vector). To construct Pep7.1, pNLF1-secNluc was used as a vector backbone and linearized using Eco*RI*. PCR cloning was used to clone the human IL-27elasti fragment from pUNO1-hIL27(ebi3p28) (InvivoGen, San Diego, CA) with a 3’ insertion of a sequence encoding peptide linker (G4S). The other pair of vectors, used for in vivo studies, were constructed using the pORF9 (plasmid open reading frame 9) as the vector backbone (Invivogen). pORF9 was linearized using *XcmI* and *NheI*. PCR cloning was used to clone the mouse IL-27elasti fragment from pUNO1-mIL-27(ebi3p28) (InvivoGen) to construct pORF9.mIL27. The empty vector (control) was named pORF9-0. All vectors were purified using GeneJET plasmid Maxiprep Kit (Thermo Scientific) according to the manufacturer’s protocol.

### Human adipose stromal cells IL-27 conditioned media (CM)

To generate IL-27-expressing-human ASC (hASC) for our gene delivery therapy, hASC were transfected via electroporation using a Neon™ transfection system (Thermo Fisher, Model MPK5000). For electroporation, ASC were first trypsinized, followed by a one-time wash with 1X PBS (Corning). hASC were re-suspended in Buffer R of the Neon transfection system at a concentration of 4 × 10^5^ cells per 100 µL of Buffer R. A total of 5 µg of plasmid DNA was added to the re-suspended cells. The plasmids used to transfect the hASC were either pNLF1-secNluc (empty vector) or Pep7.1 (human IL-27 vector) for our in vitro studies, and pORF9-0 (empty vector) or pORF9.mIL27 (mouse IL-27 vector) for our in vivo studies. To facilitate the detection of successful transfection, hASC were always co-transfected with a 10% ratio of GFP plasmid (pDr5.GFP2, Invivogen) for visualization using an inverted fluorescent microscope (Olympus IX71) (Suppl. Fig. S2A). Electroporation was performed with the following optimized conditions: 1400 V, 10 ms pulse width, and 3 pulses. Transfected cells were immediately seeded onto a fibronectin-coated 6-well plate. After an overnight incubation, cells were visualized using an inverted fluorescent microscope, and percent (%) viability (> 70%) was determined using trypan blue. The plasmid-containing medium was aspirated and replaced with ASC medium to allow cells to recover overnight. Following recovery, the medium was replaced with Opti-MEM™ I Reduced Serum Medium (Gibco) and 1% Anti-Anti to promote IL-27 secretion from the transfected cells. After 24 h of incubation with the IL-27 transfected hASC, the OptiMEM was considered ‘conditioned media’ (CM). CM was collected every 24 h up to 48 h and stored at -80 °C until use in experiments requiring CM. hASC used for in vivo studies were those that had been incubated with OptiMEM for 24 h. By gene expression analysis (qPCR), the values of subunits IL27p28 and EBI3 were significantly upregulated in ASC transfected with plasmids containing the IL27 cDNA (Suppl. Fig. S2B). Based on previous studies from our group, we expect expression levels to be ~ 10–300 fold [18] and 80-2000 pg/ml of cytokine expression in the ASC CM ( [19]– [20]).

### In vitro monoculture LPS stimulation

Lung epithelial cells (A549 or BEAS-2B) were seeded at a concentration of 1.5 × 10^5^ cells per well in a 6-well plate. Monocytes (THP-1) were seeded at a concentration of 5 × 10^5^ cells per well in a 6-well plate. Cells were immediately treated with 5 ng/mL of phorbol myristate 13-acetate (PMA) to stimulate differentiation. After 24 h, and once cell attachment was confirmed, the PMA-containing medium was removed. The medium was then replaced with the respective complete growth medium, and the cells were allowed to recover until the next day. hASC were transfected, seeded, and allowed to recover according to the previously outlined protocol. Following seeding, differentiation, or transfection recovery for lung epithelial cells, THP-1 cells, or hASC, cells were treated with lipopolysaccharide (LPS; *E. coli* O111:B4) (Sigma-Aldrich) at the noted concentration for 24 h.

To determine the effects of hASC CM on lung epithelial or monocytes, BEAS-2B or PMA-differentiated THP-1 cells were stimulated with hASC CM at varying concentrations, simultaneously with LPS administration. The concentrations of hASC CM tested in this project included 1:1, 1:5, and 1:10, with 1:1 being the highest concentration (1 part CM, 1 part growth medium) and 1:10 being the lowest (1 part CM, 9 parts growth medium).

Following 24 h LPS stimulation, all cells were washed once with 1X PBS, trypsinized, and centrifuged at 1500 rpm for 5 min. The supernatant was removed, and the cell pellet was stored at -80 °C until further RNA processing.

### In vitro co-culture LPS stimulation

Monocytes (THP-1) were seeded at a density of 5 × 10^5^ cells/well in a 6-well, 0.4 μm polycarbonate membrane transwell insert (Corning) and differentiated with 5 ng/ml of PMA. The next day, BEAS-2B cells were seeded in a separate 6-well plate at a seeding density of 1 × 10^6^ cells/well. On the same day, the PMA-containing medium in the THP-1 wells was removed and replaced with complete growth media to allow the cells to recover until the next day. Following the recovery period, the inserts containing THP-1 cells were placed in the 6-well plates containing BEAS-2B cells to simulate a co-culture system. The old medium was replaced from both the insert and the bottom well and replaced with Opti-MEM supplemented with 1% Anti-Anti, with or without LPS (1000 ng/mL), and/or hASC CM (with or without human IL-27). After 24 h, all cells were washed once with 1X PBS, trypsinized, and centrifuged at 1500 rpm for 5 minutes. The supernatant was removed, and the cell pellet was stored at -80 °C until further RNA processing.

### In vivo LPS-induced ARDS model

A total of 58 male C57BL/6 mice (8–10 weeks, 20–25 g) were obtained from Purdue Transgenic and Genomic Editing Facility or Jackson Labs, with each mouse considered an experimental unit. Mice were handled in accordance with a study protocol approved by the Purdue Animal Care and Use Committee (PACUC). All mice were housed in the animal facility with a 12 h dark light cycle and *ad libitum* access to food and water. Any animals that were excluded from the study were either due to premature death following LPS instillation or technical error during BALF collection, which prevented collection of accurate and consistent data. The sample size was calculated based on LaMorte’s Power Calculations (https://www.bu.edu/research/forms-policies/iacuc-sample-size-calculations/) utilizing values from a previous drug repurposing acute lung injury study (mean 1 = 1.75 ± 0.1; mean 2 = 1.4 ± 0.25) [21]. We determined that at least *n* = 5 is required per experimental group to achieve a power of 80% with a p-value of 0.05.

At the start of the procedure, mice were anesthetized with a combination of ketamine (Patterson Veterinary, Loveland, CO) and xylazine (Patterson Veterinary) at doses of 100 and 10 mg/kg, respectively. To induce an LPS-induced ARDS model, LPS (*E. coli* O111:B4) was resuspended in 1X PBS to a concentration of 2000 µg/mL and 5 µg/g of body weight was administered per mouse. The study was performed by two investigators. Mice were randomly assigned to different treatment groups by the first investigator, ensuring that the average weight across different groups was comparable, while treatment administration was performed solely by the second investigator to minimize variability and bias. The mice were intratracheally instilled with either 1X PBS (control) or LPS. To test the efficacy of our treatment, either 50 µL of 1X PBS (control), 5 × 10^5^ hASC with empty vector, or 5 × 10^5^ hASC with IL-27 vector were instilled per mouse at 24 h post LPS instillation, followed by an infusion of at least 200 µL of air using a syringe to ensure an even distribution across the lobes of the lungs. All animals were sacrificed at 72 h after LPS instillation, unless otherwise stated. We then collected bronchoalveolar lavage fluid (BALF), serum, lung tissue, and organ tissues from each animal for further analysis and to determine the efficacy of our therapeutic.

There were two types of intratracheal instillation routes that were compared, i.e., invasive and non-invasive. For the invasive intratracheal instillation, the anesthetized mouse was shaved over the trachea, and a skin incision was made over the trachea with a scalpel blade. The trachea was exposed with the aid of tweezers, and a 27G tuberculin syringe was inserted parallel to it to instill either 1X PBS or LPS [22]. Following injection, the incision site was sutured, and the mouse was given 20 mg/kg carprofen (Rimadyl, Zoetis, Parsippany, NJ) subcutaneously at 0 h and 24 h post-surgery to reduce pain. For the non-invasive intratracheal instillation, the anesthetized mouse was suspended in the supine position by its incisors, using a tight rubber band secured on a 45°-bent plexiglass frame [23]. A light source (LED) was positioned over the mouse’s throat to illuminate the tracheal opening. Once the tracheal opening was located, a 22G endotracheal catheter (Kent Scientific, Torrington, CT) was inserted. The work has been reported in line with the ARRIVE guidelines 2.0. To avoid potential confounding effects from carprofen, a non-steroidal anti-inflammatory drug commonly administered post-surgery, we opted to use the non-invasive intratracheal instillation method for delivering LPS. This approach eliminates the need for surgical procedures and associated analgesics, allowing for a more accurate assessment of inflammatory responses. All subsequent experiments involving IL-27 ASC therapy were conducted using this non-invasive IT model.

### Serum collection

At either 24–72 h post-LPS instillation, mice were first anesthetized with ketamine and xylazine at doses of 100 and 10 mg/kg, respectively. Mice were then sacrificed under deep anesthesia via cervical dislocation, and blood was immediately collected via cardiac puncture. The collected blood was allowed to clot at room temperature (RT) for 20 min, then centrifuged at 2,000 x g for 10 min to collect the serum. The serum was kept at -80 °C until further analysis.

### Cytokine array

Serum derived from LPS-induced mice treated with either PBS, ASC, or IL27 ASC (*n* = 4 per sample group) was assayed for a panel of chemokines/cytokines by Eve Technologies (Calgary, Alberta, Canada) to analyze levels of Eotaxin, G-CSF, GM-CSF, IFN-γ, IL-1α, IL-1β, IL-2, IL-5, IL-6, IL-9, IL-12p40, IL-13, IL-15, IP-10, KC, LIX, M-CSF, MCP-1, MIG, MIP-1α, MIP-1β, MIP-2, MIP-3α, RANTES, TNF-α, TNF-β, IL-17A, IL-17E, IL-17F, IL-22, IL-23, IL-27, and IL-28B. Multiplexed quantification of mouse cytokines/chemokines was performed using Luminex xMAP technology, a fluorescent bead-based multiplexed immunoassay system, detected by the Luminex™ 200 system (Luminex, Austin, TX, USA). A standard curve was generated to quantify levels of cytokines/chemokines present in the serum.

### Bronchoalveolar lavage fluid collection and analysis

The bronchoalveolar lavage fluid (BALF) was immediately collected following blood collection. The right main bronchus was first ligated prior to the BALF collection to avoid leakage of PBS into the right lung and minimize cell loss, thus preserving the right lung condition for subsequent gene expression and/or histology analysis. The lavages were performed by injecting and aspirating 1 ml of PBS twice before collecting the BALF. The collected BALF was then centrifuged at 300 x g for 7 min at 4 °C to obtain a cell pellet. The BALF supernatant was collected and stored at -80 °C to be used for assessing total protein concentration. Total protein concentration was analyzed using the Pierce BCA Protein Assay Kit (Thermo Scientific). The cell pellet was resuspended in 200 µL of 1X PBS. A small fraction of the resuspended cell pellet was used to estimate the total cell count using a hemocytometer.

### RNA isolation

Half of the right lung tissue was preserved in RNAlater™ Stabilization solution (Invitrogen, Carlsbad, CA) at 4 °C overnight, or for up to one week, according to the manufacturer’s protocol, to ensure RNA quality was not compromised. The lung tissues were then removed from RNAlater™ and stored at -80 °C. Total RNA was isolated from 15 to 18 mg of these lung tissues. The lung tissues were then added to 600 µL of RLT buffer, supplemented with 10 μL of β-Mercaptoethanol (MP Biomedicals, Santa Ana, CA), and homogenized using a PRO200 homogenizer (MidSci, ValleyPark, MO) in three pulses of 10–15 s each at mid-power (setting 3). For cell pellets collected from in vitro experiments, 350 µL of RLT buffer was added to each sample and homogenized by passing the cells through a 27G tuberculin syringe three times. Lysates collected from either in vitro or in vivo experiments were processed using the Qiagen RNeasy kit (Qiagen, Germantown, MD) and eluted in 50 µL of Ultrapure DNAse/RNase-free distilled water (Invitrogen).

### Reverse transcription quantitative polymerase chain reaction (RT-qPCR)

Following RNA isolation, reverse transcription was performed on 0.5 µg of RNA per sample using amfiRivert Platinum cDNA Synthesis Master Mix (GenDepot, Katy, TX). Once complementary DNA (cDNA) was obtained, RT-qPCR was performed by mixing 1 µL of cDNA, 2X KAPA SYBR Fast qPCR Master Mix (Roche, Indianapolis, IN), and 1 µM forward and reverse primers of the target genes. Primers used for RT-qPCR are listed in Table 1, with GAPDH serving as an endogenous control. The reaction was performed using the ViiA7 Real-Time PCR system and QuantStudio3 (Thermo Fisher Scientific) with the following conditions: 95 °C for 3 min, followed by 40 cycles of 95 °C for 3 s, 60 °C for 30 s, and 72 °C for 19 s. Data acquisition was performed using QuantStudio 3 software (Thermo Fisher Scientific).

**Table 1:** List of primers used for RT-qPCR

Target gene	Sequence
Human GAPDH	Forward: 5’ – ACAACTTTGGTATCGTGGAAGG – 3’
	Reverse: 5’ – GCCATCACGCCACAGTTTC – 3’
Human IL-8	Forward: 5’ – ACTGAGAGTGATTGAGAGTGGAC – 3’
	Reverse: 5’ – AACCCTCTGCACCCAGTTTTC – 3’
Human IL-1β	Forward: 5’ – AAGTACCTGAGCTCGCCAGTGAAA – 3’
	Reverse: 5’ – TTGCTGTAGTGGTGGTCGGAGATT – 3’
Human IL-6	Forward: 5’ – AGTGCCTCTTTGCTGCTTTCACAC – 3’
	Reverse: 5’ – AGCCACTCACCTCTTCAGAACGAA – 3’
Human TNF	Forward: 5’ – GAGGCCAAGCCCTGGTATG – 3’
	Reverse: 5’ – CGGGCCGATTGATCTCAGC – 3’
Human NFĸB1	Forward: 5’ – GTGGTGCCTCACTGCTAACT – 3’
	Reverse: 5’ – GGATGCACTTCAGCTTCTGT – 3’
Human EBI3	Forward: 5’ – TCATTGCCACGTACAGGCTC – 3’
	Reverse: 5’ – GGGTCGGGCTTGATGATGTG – 3’
Human IL-27p28	Forward: 5’ – GAGGGAGTTCACAGTCAGC – 3’
	Reverse: 5’ – GCAGGAGGTACAGGTTCAC – 3’
Mouse GAPDH	Forward: 5’ – AGGTCGGTGTGAACGGATTTG – 3’
	Reverse: 5’ – TGTAGACCATGTAGTTGAGGTCA – 3’
Mouse IL-1β	Forward: 5’ – GAAATGCCACCTTTTGACAGTG – 3’
	Reverse: 5’ – TGGATGCTCTCATCAGGACAG – 3’
Mouse IL-6	Forward: 5’ – ATCCAGTTGCCTTCTTGGGACTGA – 3’
	Reverse: 5’ – TAAGCCTCCGACTTGTGAAGTGGT – 3’
Mouse TNF	Forward: 5’ – CAGGCGGTGCCTATGTCTC – 3’
	Reverse: 5’ – CGATCACCCCGAAGTTCAGTAG – 3’
Mouse IL-10Ra	Forward: 5’ – CAGGCGGTGCCTATGTCTC – 3’
	Reverse: 5’ – CGATCACCCCGAAGTTCAGTAG – 3’
Mouse NFĸB	Forward: 5’ – ATGGCAGACGATGATCCCTAC – 3’
	Reverse: 5’ – TGTTGACAGTGGTATTTCTGGTG – 3’
Mouse EBI3	Forward: 5’ – CGCTCCCCTGGTTACACTG – 3’
	Reverse: 5’ – CCACGGGATACCGAGAAGC – 3’
Mouse IL-27p28	Forward: 5’ – CCGAAGTGTGGTAGCGAGG – 3’
	Reverse: 5’ – ATCTTCCCAATGTTTCCCTGAC – 3’

### RNA sequencing analysis

Total RNA was sequenced by LC Sciences (Houston, TX, USA) with analysis support. Prior to sequencing, total RNA quantity and purity were verified with an Agilent 2100 Bioanalyzer to ensure a minimum RNA Integrity Number (RIN) above 7.0. A poly(A) RNA sequencing library was then prepared following Illumina’s TruSeq-stranded-mRNA sample preparation protocol and purified using two rounds of oligo-(dT) magnetic beads purification. Following the purification, poly(A) RNA was fragmented using divalent cation buffer at elevated temperature. Quality control and quantification of the sequencing library was verified using an Agilent Technologies 2100 Bioanalyzer High Sensitivity DNA Chip. Paired-ended sequencing was performed on Illumina’s NovaSeq 6000 sequencing system.

For transcript assembly, reads that contained adaptor contamination, low quality bases and undetermined bases were removed using Cutadapt [24] and Perl scripts. Sequence quality was verified using FastQC (http://www.bioinformatics.babraham.ac.uk/projects/fastqc/) and mapped to the *Mus musculus* (mouse) genome using HISAT2 [25]. Assembly of mapped reads from each sample was performed with StringTie [26] and then merged to reconstruct a comprehensive transcriptome using perl scripts and gffcompare. StringTie and ballgown (https://www.bioconductor.org/packages/release/bioc/html/ballgown.html) were employed to estimate the expression levels of all transcripts. The mRNA expression levels were calculated using StringTie and reported in Fragments per Kilobase of transcript per Million mapped reads (FPKM). To analyze differential gene expression of mRNAs between two different groups, the R package DESeq2 [27] was used while the R package edgeR [28] was used for analysis between two samples. We considered mRNAs with a p-value < 0.05 and absolute fold change ≥ 1.5 as differentially expressed mRNAs.

To gain insights into biological processes and pathways associated with the identified differential gene expressions, we utilized Metascape [29], a web-based tool for gene annotation and functional enrichment analysis. To perform the functional enrichment analysis, lists of differentially expressed genes were uploaded to the Metascape website (https://metascape.org/), in which appropriate species (Mus musculus) was specified and desired gene annotation resources and pathway databases were selected. Here, all annotations terms were selected, membership category included all functional sets, pathways, and structural complexes. For enrichment analysis (custom) settings, a minimum overlap of 3 and p of 0.05 were selected for pathways and processes, with the PPI enrichment combined (all) databases. Also used was g:Profiler, a tool for functional profiling of transcript lists (https://biit.cs.ut.ee/gprofiler/) to further investigate regulatory mechanisms, for instance, to identify enriched transcription factors among upregulated transcripts, applying the g: GOSt module with multiple testing correction to highlight potential upstream regulators.

### Immune cell profiling using RNA-sequencing data

TIMER2.0 [30] was used to estimate the immune composition within the lung microenvironment following various treatments, using transcripts per million (TPM) data matrices as an input. While TIMER2.0 is commonly used for analysis of immune infiltrates in the context of tumors, it uses the immunedeconv [31] package that integrates several algorithms that provide cell type signatures derived from any complex tissues, as recently demonstrated in the literature [32].

### Histology

The other half of the right lung, along with other organ tissues (i.e., heart, kidney, liver, spleen, and intestines), were fixed in 10% formalin (ThermoFisher) for 24 h. The next day, formalin was removed, and all tissues were stored in 70% ethanol until further processing. The tissues, kept in tissue cassettes, were processed by the Purdue Histology Research Laboratory for H&E staining. Histological images were captured using an Olympus BX51 and Olympus BX53. To quantify the extent of lung injury between different treatment groups, the histological images were assigned a score based on the lung injury scoring system described by Matute-Bello et al. [33]. Here, at least 20 random high-power fields (400X total magnification) were scored blindly. Each histology image was graded accordingly based on the number of neutrophils in the alveolar space, the number of neutrophils in the interstitial space, presence of hyaline membranes, presence of proteinaceous debris in the airspaces, and thickness of alveolar septa. The proposed scoring system is both simple and accessible to laboratory researchers and pathologists, mitigating interobserver variability.

### Statistical analysis

Statistical analysis of RNAseq data was performed using DESeq2 [27]. All other analyses were conducted using Graph Pad Prism version 10 (GraphPad Software, San Diego, CA, USA). In some in vivo studies, log10 transformations were used for inter-animal variability normalization. Outcome measures were reduction in inflammation as per BALF counts and histology scores vs. control, for determining sample size numbers. Calculations to determine statistically significant differences across treatment groups were performed using t-test or ANOVA in which a p-value < 0.05 was considered significant.

## Results

### Characterization of LPS-induced monoculture cells to establish an in vitro ARDS model

To assess the ability of targeted IL-27 in reducing inflammation, we first needed to establish an in vitro model of ARDS that mimics the clinical condition. ARDS patients typically show upregulation of pro-inflammatory cytokines, particularly IL-8, IL-1β, IL-6, and TNF-α [34]. Several studies have shown that subjecting cells to LPS stimulation results in the upregulation of pro-inflammatory cytokines, mimicking the response seen in ARDS caused by pneumonia. However, the degree of response can vary depending on the cell line used [35, 36] and the *E. coli* strain from which the LPS was derived [37]. Thus, the goal of this experiment was to determine the LPS concentration for the cell lines we use and characterize their pro-inflammatory cytokine responses. We tested responses from lung epithelial cells, as the major cell type that makes up the lungs, macrophages, which are the first responders during inflammation, and human adipose derived MSC (hASC), which serve as our gene delivery carriers.

**Fig. 1:**
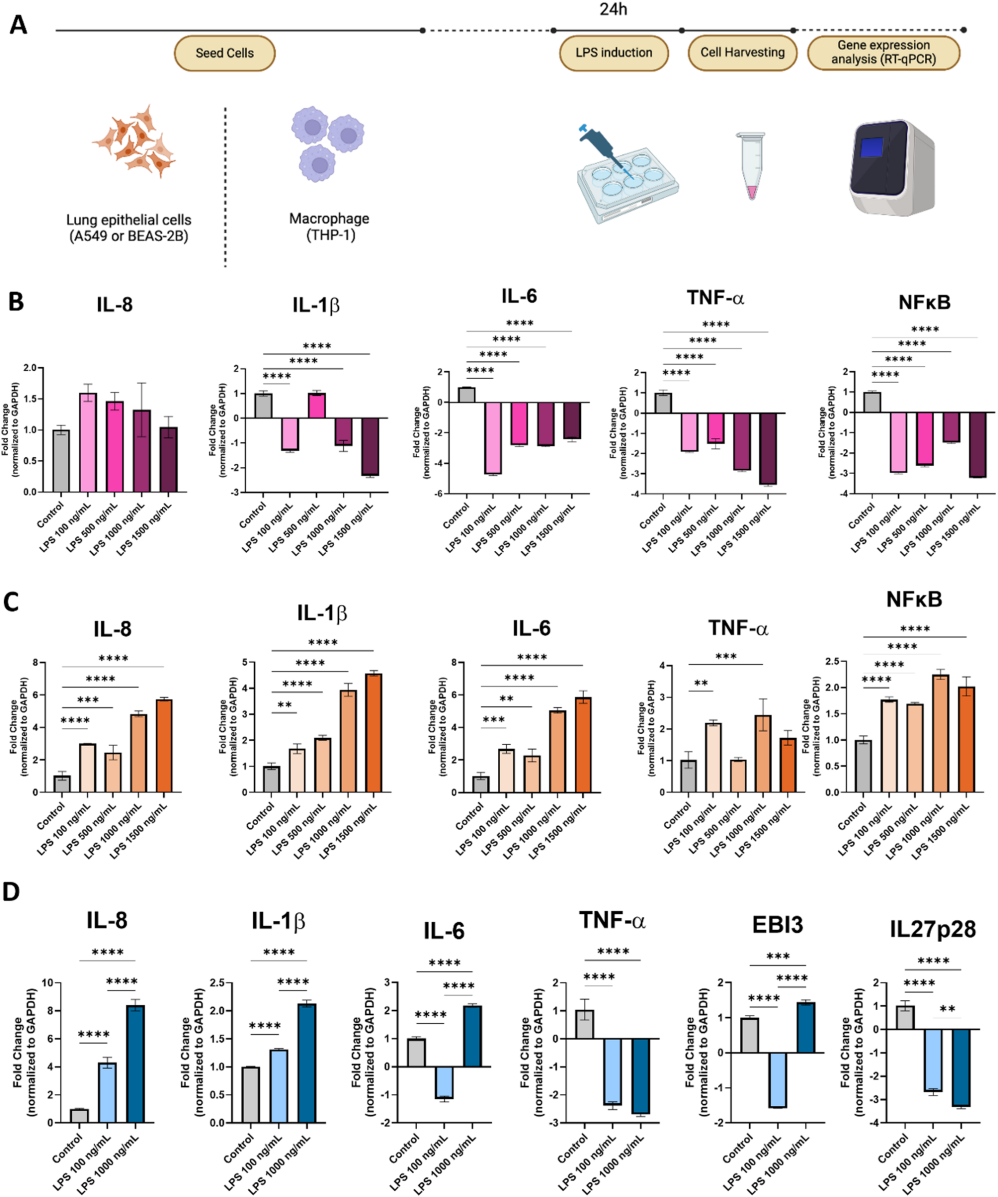
Characterization of several cell line responses in an LPS-induced in vitro ARDS model. (**A**) Overview of the monoculture LPS-induced in vitro ARDS model methodology. Different cell lines, including lung epithelial cells (A549 or BEAS-2B) or monocytes (THP-1), were seeded in a 6-well plate overnight. Monocytes were PMA-differentiated for 24 h prior to LPS induction. Cells were then treated with LPS for 24 h before the cell pellets were harvested. Varying concentrations of LPS were utilized ranging from 0-1000 ng/mL, as indicated. Gene expression was then analyzed using RT-qPCR. Created with Biorender.com. Gene expression fold change of monoculture A549 (**B**), BEAS-2B (**C**), and THP-1 (**D**) cells at varying concentrations of LPS. One-way ANOVA was conducted using GraphPad Prism and data are presented as mean ± SD. *p < 0.05, **p < 0.01, ***p < 0.001, ****p < 0.0001

The A549 cell line was the first to be investigated, as it is one of the most commonly used lung epithelial models in ARDS studies [35, 37]. Upon 24 h of LPS stimulation at varying concentrations, A549 cells were harvested, and pro-inflammatory cytokine gene expression from isolated mRNA was analyzed with RT-qPCR (Fig. 1A). While A549 showed upregulation of IL-8, this upregulation was not LPS dose-dependent (Fig. 1B). More interestingly, A549 also showed downregulation of IL-1β, IL-6, TNF-α, and NFĸB compared to control cells in response to LPS stimulation. This response may be due to the nature of A549, which is derived from lung carcinoma, and may respond differently than cells derived from healthy patients. Therefore, we conducted further characterization using BEAS-2B cells, which are derived from normal human bronchial epithelium of noncancerous origin. Upon LPS stimulation at varying doses, BEAS-2B cells showed a significant, dose-dependent upregulation of IL-8, IL-1β, and IL-6, with a 4- to 6- fold increase at a dose of 1000 ng/mL of LPS (Fig. 1C). There was also an increasing trend in TNF-α and NFĸB expression as LPS dose increased; however, there was a slight reduction in gene expression observed at the 1500 ng/mL dose, possibly due to toxicity at high LPS concentrations, as seen in previous studies [38]. Hence, BEAS-2B cells were chosen as the lung epithelial model for all future in vitro studies of LPS-induced ARDS, with 1000 ng/mL chosen as the upper dose limit for LPS.

Next, the THP-1 human monocytic cell line was used to characterize the monocyte/macrophage response to LPS stimulation. THP-1 cells were first differentiated with PMA for 24 h, followed by 24 h of LPS stimulation. Gene expression analysis by RT-qPCR showed significant upregulation of IL-8, IL-1β, and IL-6, especially at the 1000 ng/mL LPS concentration (Fig. 1D). Thus, 1000 ng/mL LPS was used for the in vitro ARDS model moving forward.

**Fig. 2:**
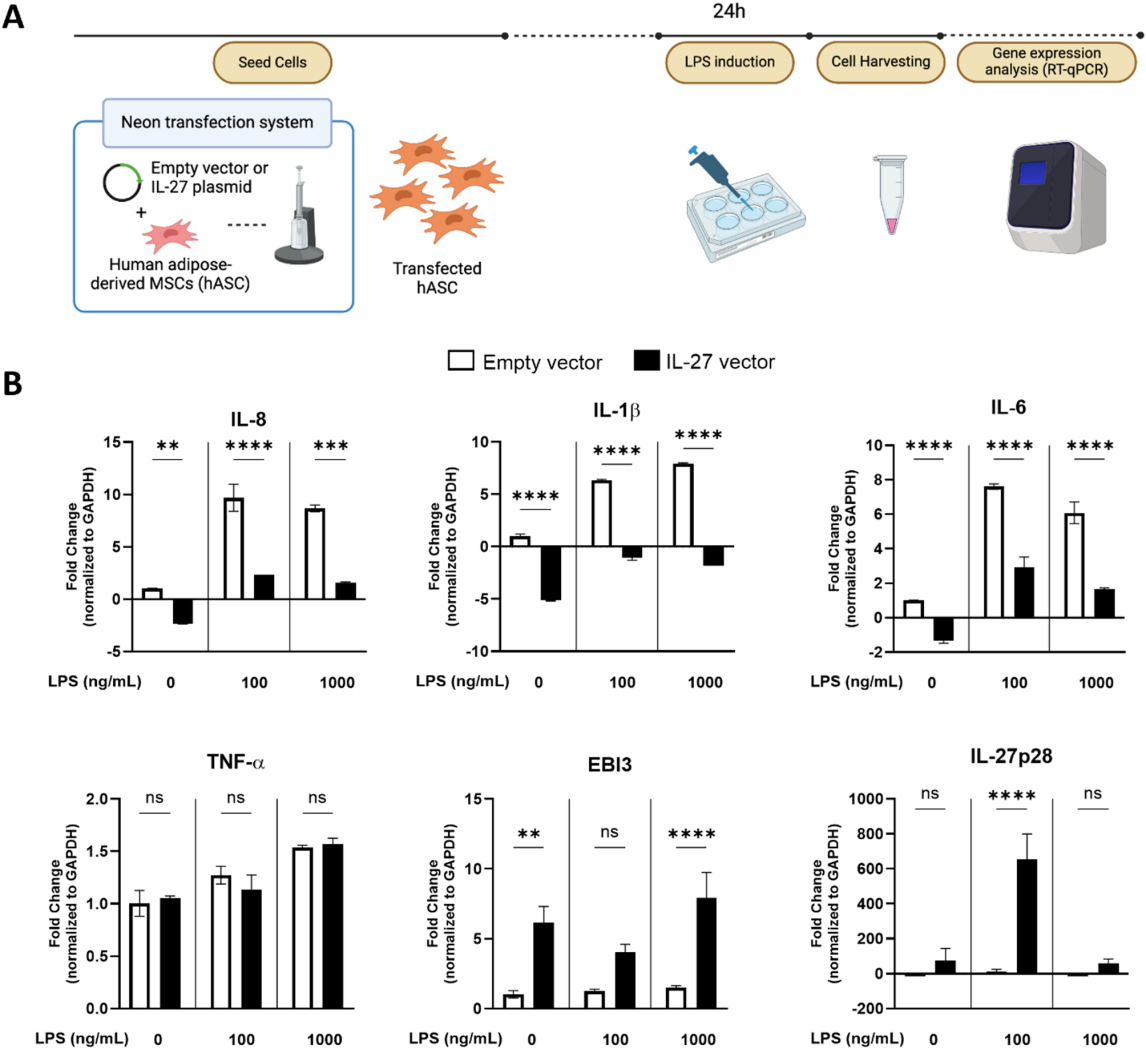
Characterization of human adipose-derived MSC (hASC) in an LPS-induced ARDS model. (**A**) Overview of the monoculture LPS-induced in vitro ARDS model methodology of transfected hASC. Prior to seeding, hASC were co-transfected with a GFP-expressing plasmid and either an empty vector or a human IL-27 vector using electroporation (Neon transfection system). Cells were allowed to recover before being induced with LPS for 24 h, and gene expression was analyzed with RT-qPCR. Created with Biorender.com (**B**) Gene expression fold change of monoculture transfected hASC in varying concentrations of LPS (0, 100, 1000 ng/mL). One-way ANOVA was conducted using GraphPad Prism and data are presented as the mean ± SD. *p < 0.05, **p < 0.01, ***p < 0.001, ****p < 0.0001

Lastly, hASC were characterized in response to LPS stimulation by first transfecting them with either an empty vector (control) or a human IL-27-expressing plasmid prior to the 24 h of LPS stimulation (Fig. 2A). Surprisingly, hASC showed upregulation of IL-8, IL-1β and IL-6 (Fig. 2B), contrary to the anti-inflammatory characteristics reported in the literature [39]. However, IL-27-expressing hASC showed a significant reduction in IL-8, IL-1β, and IL-6 compared to control groups, and this suppression was maintained even at the high LPS concentration of 1000 ng/mL. This result suggested that IL-27-expressing hASC exhibit an intrinsic anti-inflammatory response, even in the presence of LPS stimulation, and could potentially counteract the inflammatory microenvironment associated with ARDS.

### IL-27 conditioned media reduces pro-inflammatory cytokine gene expression in monocultures of lung epithelial and monocyte cell lines

To evaluate the effects of our IL-27 gene therapy in the established LPS-induced in vitro model of ARDS, conditioned media (CM) from our transfected hASC were generated as outlined in the Methods section. Monoculture lung epithelial cells (BEAS-2B) and macrophage (THP-1) were stimulated with LPS for 24 h, followed by treatment with different concentrations of control vector or IL-27 hASC CM (Suppl. Fig. S3A).

Varying concentrations of CM induced different responses in the gene expression of pro-inflammatory cytokines in LPS-induced BEAS-2B and THP-1 cells (Suppl. Fig. S3B&C). In BEAS-2B cells receiving CM 1:1, there was a significant downregulation of IL-1β, significant upregulation of IL-17A, and a slight upregulation of IL-8 and IL-6 in the IL-27 groups compared to the control groups. In contrast, in the more diluted CM groups (1:5 and 1:10), IL-27 CM was able to maintain or slightly reduce, and in some cases, significantly reduce, the gene expression of pro-inflammatory cytokines compared to the control CM. It is possible that diluting the CM also dilutes metabolites that affect cytokine production [40]. Similarly, in LPS-induced THP-1 cells, we also observed reduced production of pro-inflammatory cytokines upon addition of IL-27 CM compared to control CM, depending on the concentration of CM used. The greatest significant reduction in expression across the genes tested was seen with the 1:5 IL-27 CM treatment, reflected in the reduced expression of IL-8, IL-1β, TNF-α, and NFĸB. Based on the results from both LPS-induced BEAS-2B and THP-1 cells, we chose to use a 1:5 CM concentration to test the effects of our therapy in a co-culture model.

### IL-27 conditioned media reduces pro-inflammatory cytokine gene expression in a lung epithelial-macrophage co-culture model of LPS-induced ARDS

The pathogenesis of ARDS involves interactions between many tissues in the lung microenvironment, and thus, any immunological response is a culmination of crosstalk between these tissues. Therefore, a lung epithelial-macrophage co-culture model was used to better mimic this microenvironment and serve as an in vitro model for ARDS induced by LPS.

The co-culture assay was performed in a 6-well transwell with 0.4 μm membrane that prevents cell migration but allows cell secretions to permeate through, facilitating cell-cell communication. BEAS-2B and THP-1 cells were seeded at a ratio of 2:1, based on the estimated lung-to-monocyte ratio in human lung composition [41].

**Fig. 3:**
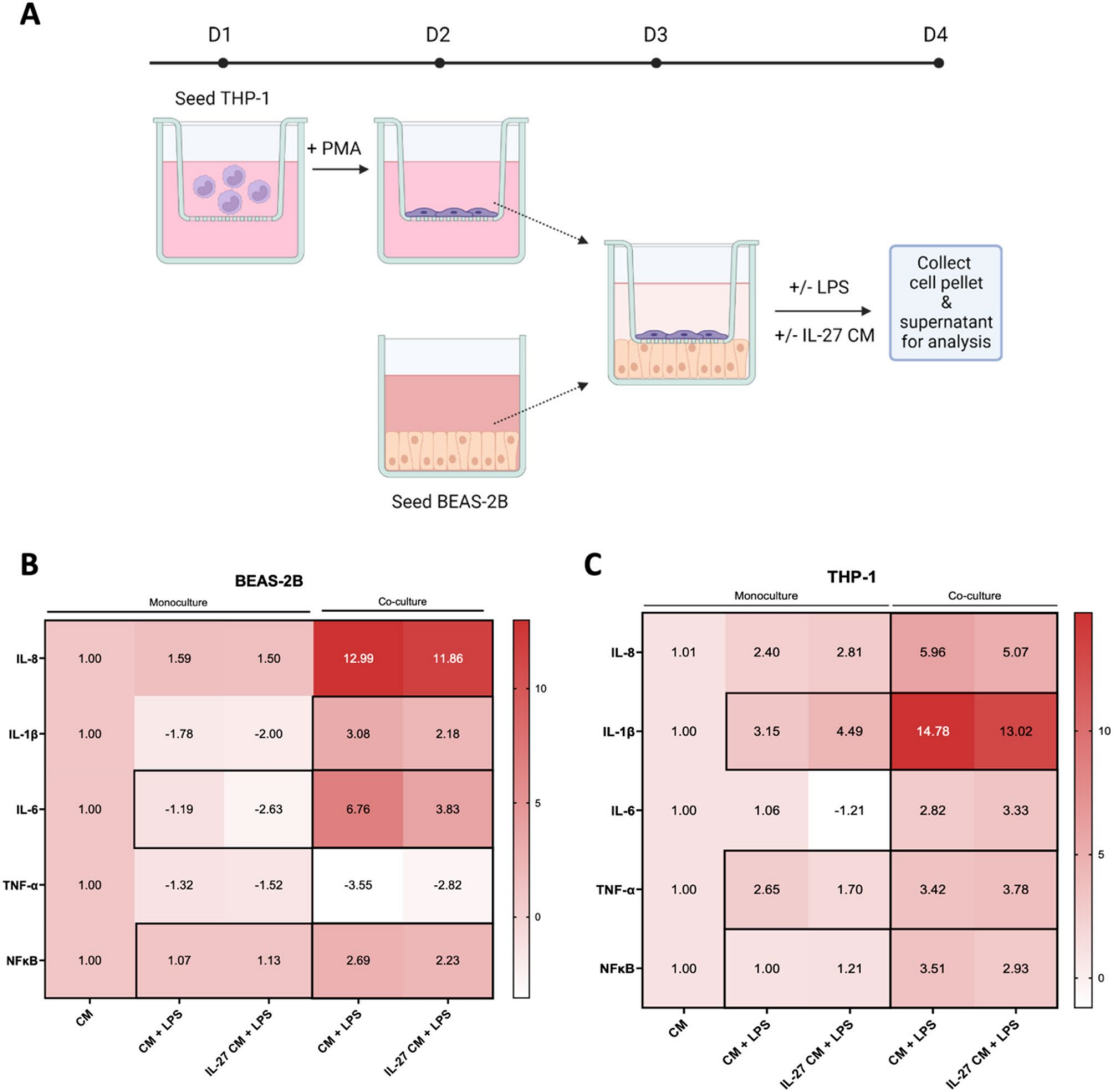
Effects of IL-27 hASC CM on LPS-induced lung epithelial and macrophage co-culture. (**A**) Methodology overview of LPS-induced in vitro ARDS co-culture model. THP-1 cells were PMA-differentiated prior to co-culture with BEAS-2B cells and treatment with LPS (1000 ng/mL) and IL-27 hASC CM (1:5) for 24 h. Gene expression heatmap of BEAS-2B (**B**) and THP-1 (**C**) cells in monoculture and co-culture, stimulated with LPS and/or IL-27 hASC CM. Gene expression fold changes were normalized to the endogenous control (GAPDH). Upregulated genes are represented with red, and downregulated genes in white. One-way ANOVA was conducted using GraphPad Prism, and data are presented as the mean. Black borders around two groups denote statistical significance of at least *p* < 0.05

In the monoculture of BEAS-2B cells, similar to our previous results, LPS-induced BEAS-2B cells showed significant downregulation of IL-6 upon IL-27 CM treatment, as well as significant upregulation of NFĸB. Also, there was a trend toward downregulation of IL-8, IL-1β, and TNF-α gene expression (Fig. 3B). In the THP-1 monoculture, TNF-α was significantly downregulated, and IL-6 expression was reduced slightly (Fig. 3C). Meanwhile, IL-1β and NFĸB gene expression were significantly upregulated, though with less than a 2-fold change. The differences in gene expression trends between these monoculture groups and the previous experiment might be due to differences in cell numbers used. Nevertheless, in both BEAS-2B and THP-1 co-culture groups, fold changes in gene expression were amplified (Fig. 3B&C). In the BEAS-2B co-culture, significant reductions of IL-1β, IL-6, and NFĸB were observed, with a slight reduction in IL-8 expression in the presence of IL-27. Similarly, in the THP-1 co-culture significant downregulation of IL-8, IL-1β, and NFĸB gene expression was observed upon IL-27 CM treatment. These results showed that the presence of IL-27 in the CM was able to significantly downregulate the expression of several pro-inflammatory cytokine genes compared to the control CM alone.

### Establishing an in vivo model of ARDS

Promising results from the in vitro studies showed us the potential of using IL-27 hASC as treatment for ARDS. However, we acknowledge that our co-culture assay does not fully recapitulate the lung microenvironment, as it lacks numerous cells (endothelial cells, immune cells, stem cells, etc.) and many factors within the organism (air-lung interface, pressure, kinetics/mechanics during breathing, immune cell responses, crosstalk between various cells). Moreover, it is possible that continuous secretion of IL-27 from living hASC may enhance the therapeutic efficacy compared to IL-27 hASC CM. Thus, to better understand the effects of IL-27 hASC, we first needed to establish an in vivo model of ARDS.

To determine that we had successfully induced an ARDS model in vivo, several measurements of relevant features were verified according to the criteria described by Matute-Bello et al. [33]. The key features focused on in this study included histological evidence of tissue injury (based on standardized histology images), increased protein concentration and the absolute number of cell infiltrates in the bronchoalveolar lavage fluid (BALF), and the elevated levels of pro-inflammatory cytokines in lung tissue.

**Fig. 4:**
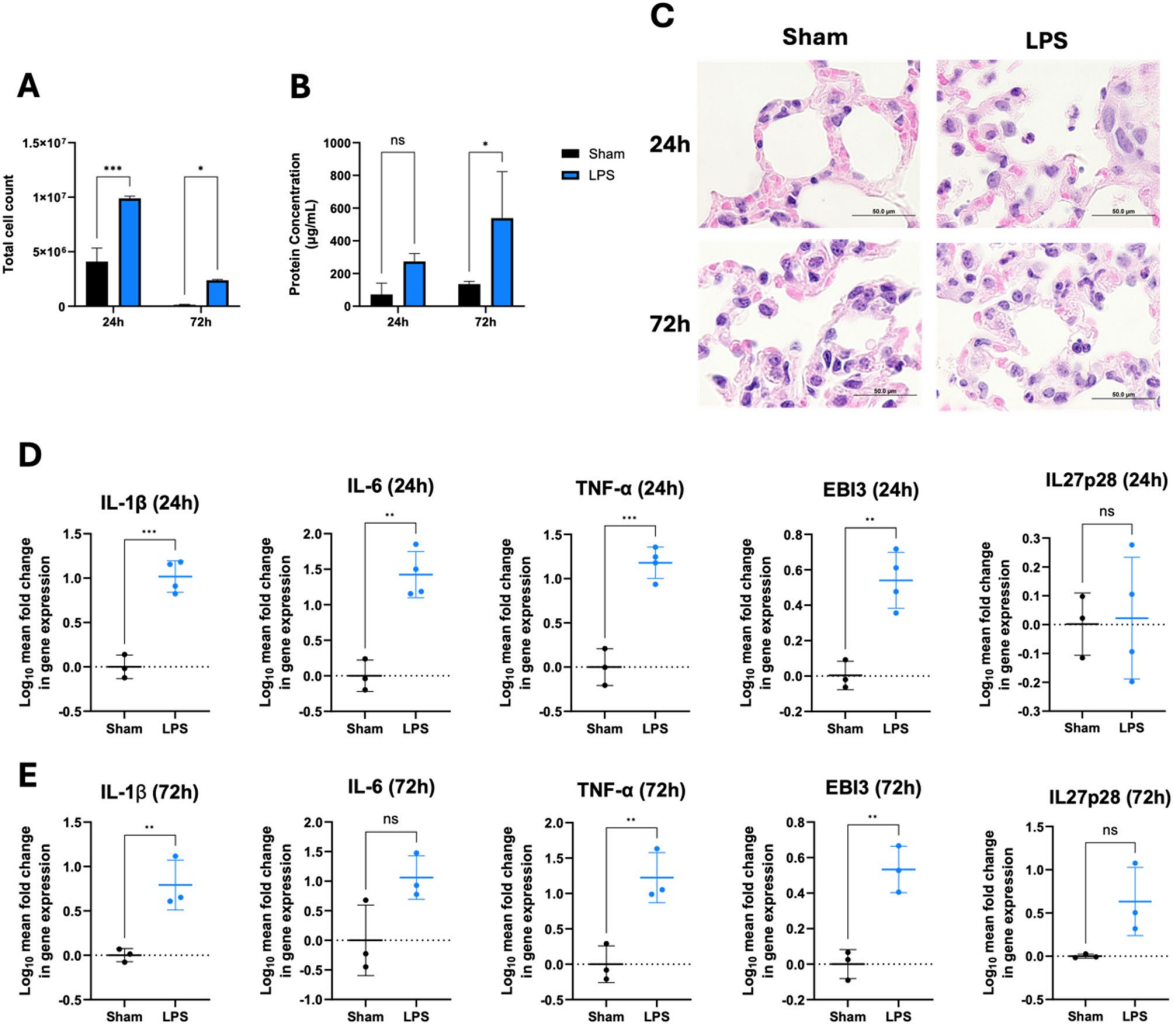
Verification of in vivo LPS-induced ARDS model. Male C57BL/6 mice were subjected to a direct, invasive intratracheal injection of PBS or LPS (5 mg/kg) and sacrificed at 24 h and 72 h post-injection. (**A**) Total cell count from bronchoalveolar lavage fluid (BALF) at 24 h (PBS, *n* = 2; LPS, *n* = 3) and 72 h post injection (PBS, *n* = 3; LPS, *n* = 2). Two-way ANOVA and uncorrected Fisher’s LSD, with single pooled variance was performed using GraphPad Prism. (**B**) Total protein concentration from BALF at 24 h and 72 h. (**C**) H&E staining of lung tissues at 100X magnification. Gene expression fold change of pro-inflammatory cytokines (IL-1β, IL-6, TNF-α) and IL-27 subunits (EBI3 and IL27p28) at 24 h (**D**) and 72 h (**E**) post-injection (*n* = 3 per group). Gene expression is presented as log10. Unpaired t-test with Welch’s correction was performed using GraphPad Prism. Data are presented as mean ± SEM.*p < 0.05, **p < 0.01, ***p < 0.001

To generate our in vivo ARDS model, male C57BL/6 mice were intratracheally injected with either 1X PBS (sham) or 5 mg/kg of LPS through a direct, invasive method. We investigated the responses after 24 h and 72 h post-LPS stimulation. At 24 h post-LPS stimulation, the LPS group showed a significant increase in the total number of cells present in the BALF (Fig. 4A). By 72 h, the total number of cells present in the BALF was significantly reduced, but a significant difference remained between the sham and LPS groups. The total BALF protein concentration at the 72 h timepoint was also significantly higher in the LPS group compared to the sham group (Fig. 4B). Histological analysis showed greater cell infiltration and a reduced area of alveolar sacs in the LPS group compared to the sham group, even at the 72 h timepoint (Fig. 4C). Gene expression analysis of mRNA isolated from lung tissues also indicated significant upregulation of pro-inflammatory cytokines, namely IL-1β, IL-6, and TNF-α, in the LPS group compared to the sham group (Fig. 4D&E). The 24 h timepoint had a higher magnitude of fold change in gene expression (10-fold, 26-fold, and 15-fold for IL-1β, IL-6, and TNF-α, respectively). Nevertheless, significant differences between the sham and LPS group were still observed even at 72 h (6-fold, 11-fold, and 16-fold changes in IL-1β, IL-6, and TNF-α, respectively). Interestingly, there seems to be a delayed response in the secretion of IL-27 upon LPS stimulation. While EBI3 was significantly upregulated at both 24 h and 72 h in the LPS group compared to the sham group, IL-27 is typically not secreted unless IL27p28 is also being produced [3]. At 72 h, a 4-fold upregulation of IL27p28 expression was observed in the LPS group (Fig. 4E). This suggests that IL-27 plays a role in ARDS, and the timing of IL-27 expression and/or administration could be crucial to ameliorate ARDS.

Additionally, we generated an in vivo ARDS model using a non-invasive intratracheal instillation of LPS. Results from the BALF showed similar trends to those observed with the invasive intratracheal method, with the LPS group showing higher protein concentration compared to the sham group (Suppl. Fig. S4A&B), albeit these differences were not statistically significant. Gene expression analysis aligned with our invasive intratracheal ARDS model, with significant upregulation of pro-inflammatory cytokines (IL-1β, IL-6, and TNF-α) (Suppl. Fig. S4C). However, the magnitude of cell counts (1.76 × 10^6^ vs. 2.37 × 10^6^ cells) and gene expression levels of IL-6 and TNF-α in the non-invasive model were slightly lower than in the invasive intratracheal model. This is to be expected, as surgical procedures introduce additional injury to the organism, which in turn promotes a greater immune response that can lead to detecting significant changes in subtle differences. Despite this, non-invasive intratracheal instillation of LPS bypasses the use of carprofen, which is a non-steroidal anti-inflammatory drug that is used as a pain reliever post-surgery. In our preliminary data using invasive intratracheal instillation of LPS, we did not observe significant changes in BALF count and gene expression following the administration of our treatment; rather, there was a trend of slight upregulation in pro-inflammatory gene expression following ASC and/or IL-27 ASC therapy (Suppl. Fig. S5). We hypothesize that the interaction between this anti-inflammatory drug and the ASC and/or IL-27 ASC therapy may have impeded the efficacy of the treatment, however future exploration would be required to strongly support this claim and understand this phenomenon in greater detail. Regardless, we chose to use non-invasive intratracheal instillation of LPS and our gene therapy (IL-27 ASC) in following experiments.

### Effects of IL-27-expressing hASC in a non-invasively induced intratracheal LPS model of ARDS in vivo

**Fig. 5:**
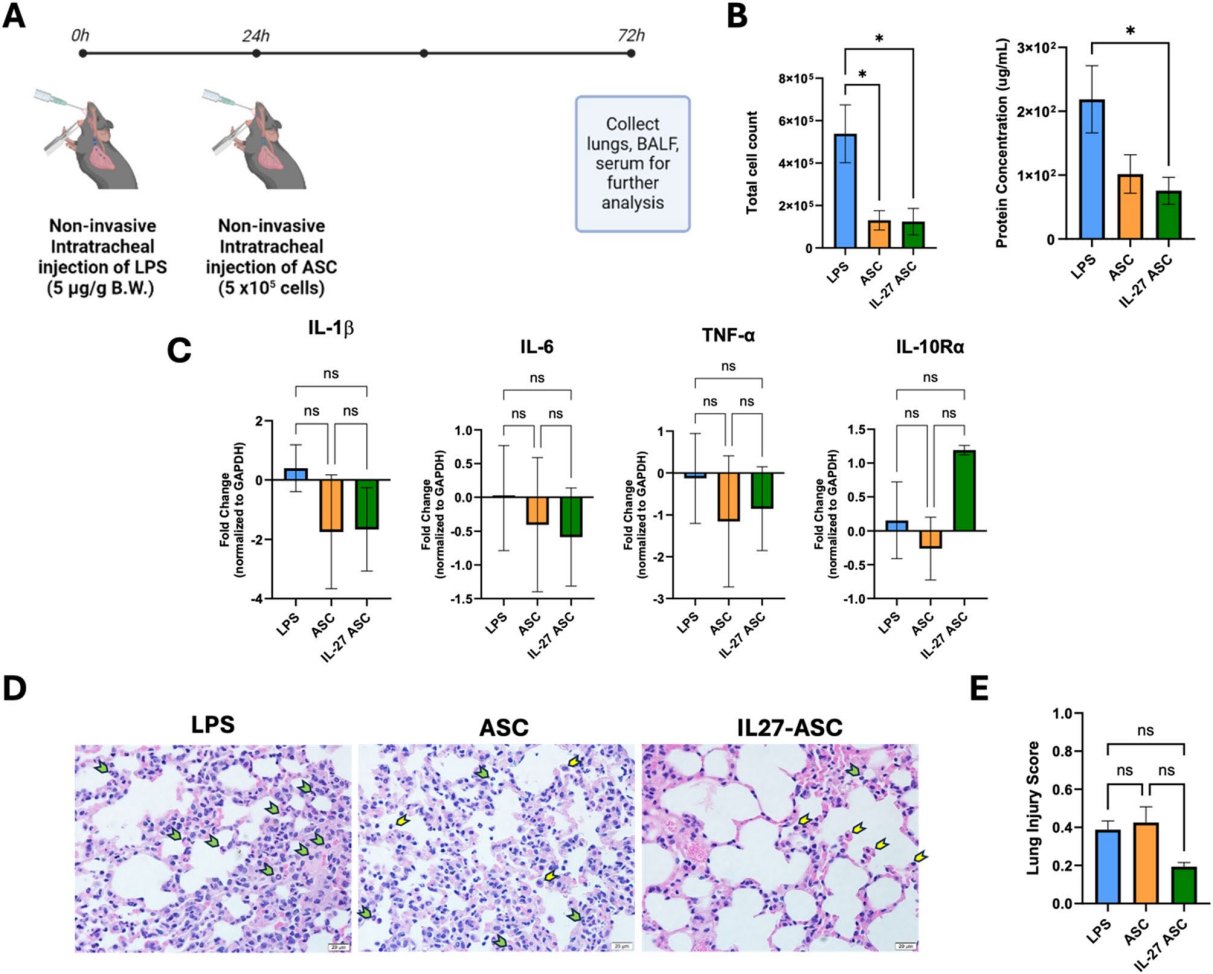
Effects of IL-27-expressing hASC administration on a non-invasive intratracheal LPS-induced in vivo ARDS model. (**A**) Methodology overview. Male C57BL/6 mice were subjected to non-invasive intratracheal injection of LPS (5 mg/kg). 24 h post LPS-induction, mice were administered PBS (*n* = 7), ASC (5 × 10^5^ cells / mouse) (*n* = 8), or IL-27 ASC (IL-27 expressing hASC) (*n* = 10) through a non-invasive intratracheal injection. Mice were sacrificed at 72 h post LPS induction. (**B**) Total cell count of BALF and total protein concentration at 72 h. (**C**) Gene expression fold change derived from mRNA extracted from mouse lung at 72 h. Unpaired t-test was performed using GraphPad Prism. Data are presented as mean ± SEM. *p < 0.05 (**D**) Representative histology images of mouse lungs at 72 h. Green arrows indicate neutrophils, while yellow arrows indicate monocytes/macrophages. (**E**) Lung injury score was calculated as the average of 20 images per sample group

To test the efficacy of our gene therapy in an LPS-induced ARDS model, we repeated our experiment using a non-invasive intratracheal instillation of LPS (5 mg/kg) in male C57BL/6 mice (Fig. 5A). 24 h following LPS instillation, either 1X PBS (LPS group), hASC (5 × 10^5^ cells/mouse) receiving an empty vector (ASC group), or IL-27 (IL-27 ASC group) were administered intratracheally (non-invasive). Results from BALF counts indicated a significant reduction in total cell infiltrates (Fig. 5B), with significantly fewer cells in both the ASC and IL-27 ASC groups compared to the LPS control. The total protein concentration in the BALF also aligned with these results, showing a decreasing trend in protein levels in both ASC groups (with and without IL-27). Interestingly, significant reduction of protein concentration was only seen in the IL-27 ASC group, not the ASC group, when compared to the LPS control. Gene expression analysis showed a similar trend, with both the ASC and IL-27 ASC groups demonstrating a reduction in pro-inflammatory cytokine (IL-1β, IL-6, and TNF-α) gene expression compared to the LPS group (Fig. 5C). The IL-27 ASC group showed a greater reduction in pro-inflammatory gene expression compared to the ASC group, albeit this was not statistically significant. Furthermore, the IL-27 ASC group showed a significant increase in IL-10 Rα gene expression compared to the ASC group. Histologically, we observed differences between the lung tissues of LPS, ASC, and IL-27 ASC groups (Fig. 5D&E), and no difference in other organs, suggesting no toxicity following the treatment administered (Suppl. Fig. S6). While PBS controls were not included in this specific analysis, future studies will incorporate matched vehicle controls and extended safety assessments. There was a trend of decreasing lung injury scores with treatment of IL-27 ASC compared to ASC (*p* = 0.0609). Even at 72 h, the LPS group still showed neutrophil infiltration within the alveolar space and interstitium. In contrast, both the ASC and IL-27 ASC groups had an overall reduction of cell infiltrates and thinner alveolar septa. We also noted that while there were fewer neutrophils in the IL-27 ASC group, more monocytes/macrophages were present. Overall, the results from this in vivo study show promise for the use of IL-27 ASC to reduce inflammation in the ARDS context.

### Cytokine/Chemokine analysis of serum

**Fig. 6:**
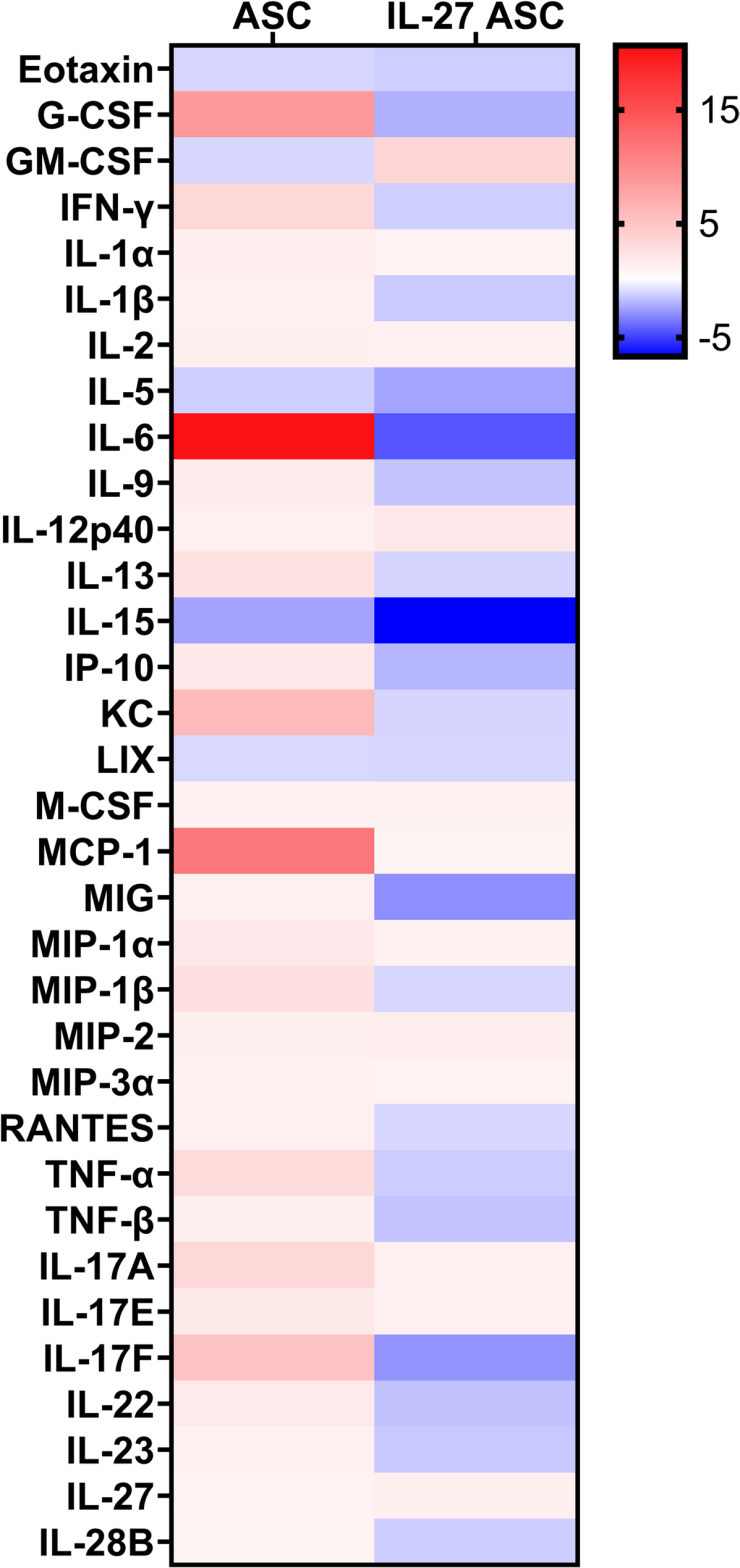
Heatmap of a multiplex cytokine/chemokine panel. Mouse serum derived from an ARDS in vivo model treated with PBS (LPS), ASC, or IL-27 ASC were analyzed for various cytokine and chemokines. Fold change was calculated relative to the control group (LPS) and presented as a heatmap, in which red indicates increased levels while blue indicates decreased levels of cytokines/chemokines relative to the control.

In addition to collecting lung tissues and BALF, we also collected serum from mice subjected to the LPS-induced ARDS model at the 72-h time point. We quantified various cytokines and chemokines in the serum using a multiplex cytokine panel. Fold changes were calculated and normalized to the control group (LPS) and presented as a heatmap (Fig. 6).

Trend-wise, we observed that the addition of ASC, with or without IL-27, to the LPS-induced ARDS model was able to decrease the levels of eotaxin, LIX, IL-5, and IL-15. IL-27 ASC also had a greater effect in reducing levels of IL-15 compared to ASC alone. This suggests that ASC, with or without IL-27 can reduce biomarkers associated with ARDS disease prognosis [42, 43].

Interestingly, we also noted that several cytokine levels were increased in the presence of ASC alone but could only be decreased in the IL-27 ASC groups. These chemokines/cytokines included G-CSF, IFN-γ, IL-6, IP-10 (CXCL10), KC (CXCL1), MCP-1, MIG (CXCL9), MIP-1β, RANTES, TNF-α, TNF-β, IL-17A, IL-17E, IL-17F, IL-22, and IL-23. As these cytokines/chemokines play a role in recruiting immune cells and promoting inflammation, the results suggest that the addition of IL-27 ASC is able to reduce immune cell migration and modulate pro-inflammatory functions that are upregulated during lung injury.

Aside from cytokines involved in the recruitment and activation of immune cells, we also observed changes in cytokines associated with lung epithelial repair, namely IL-28B and GM-CSF. IL-27 ASC was able to reduce IL-28B levels and increase GM-CSF levels. We also noted a slight increase in IL-27 levels in the IL-27 ASC group, suggesting that IL-27 production was increased, likely due to the IL-27-expressing plasmid transfected into the ASC. Overall, the serum analysis data suggested that IL-27 ASC treatment results in a reduction of chemokines/cytokines involved in immune cell recruitment to inflammatory sites while modulating cytokines that tend to be pleiotropic, thus promoting beneficial inflammation as well as tissue repair.

### Differential expression of genes in lung tissues

**Fig. 7:**
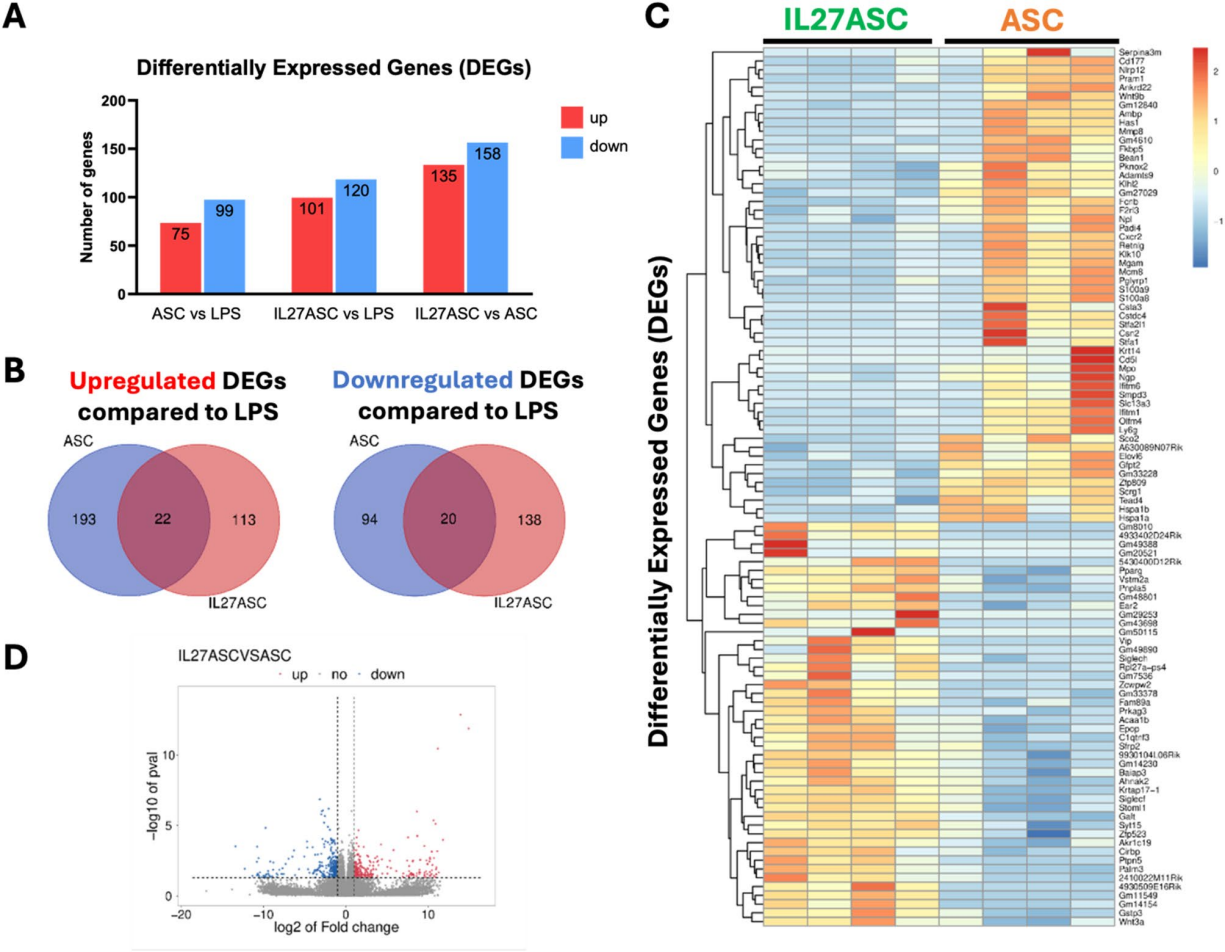
Summary of Differentially Expressed Genes derived from LPS-induced mouse lungs. Genes were considered differentially expressed genes (DEGs) if their Log2FC ≥ 1.5 or ≤-1.5 and p-value < 0.05. (**A**) Bar plots indicating the number of DEGs in different treatment groups. (**B**) Venn diagrams showing the upregulated and downregulated DEGs in ASC and IL-27 ASC groups relative to control (LPS), and the overlapping genes between them. Venn diagrams were created using the Van de Peer diagram tool [44]. (**C**) Heatmap of DEGs from LPS-induced mouse lungs treated with ASC and IL27 ASC relative to control (LPS), *n* = 4 per sample group. (**D**) Volcano plot showing DEGs in IL27 ASC vs. ASC

To further understand the mechanisms and pathways involved in the therapeutic effects of IL-27 ASC following an LPS-induced ARDS model, we conducted RNA sequencing of RNA extracted from the lungs of mice subjected to different treatments. Lung tissues were harvested 72 h post-LPS induction of the ARDS model or 48 h post-administration of various treatments (control (LPS), ASC, IL-27 ASC). Total mRNA (*n* = 4 per sample group) was sequenced and analyzed as described in the Methods section. We identified differentially expressed genes (DEGs) and represented them (Log2(FC) ≥ 1.5 or ≤ -1.5 and *p* < 0.05) relative to the control (LPS group) (Fig. 7).

Through RNA-seq analysis, we identified a total of 688 genes that were differentially expressed across the different treatment groups (LPS, ASC, and IL-27 ASC) (Fig. 7A). Of these, 580 genes were specifically expressed in the ASC and IL-27 ASC treatment groups relative to LPS (Fig. 7B). Among these, 538 genes (92.8%) were unique to a single treatment group, and 42 genes (7.24%) were common between the ASC and IL-27 ASC groups. The heatmap of the DEGs indicated distinct separation of clustering profiles between the ASC and IL-27 ASC treatments (Fig. 7C).

**Fig. 8:**
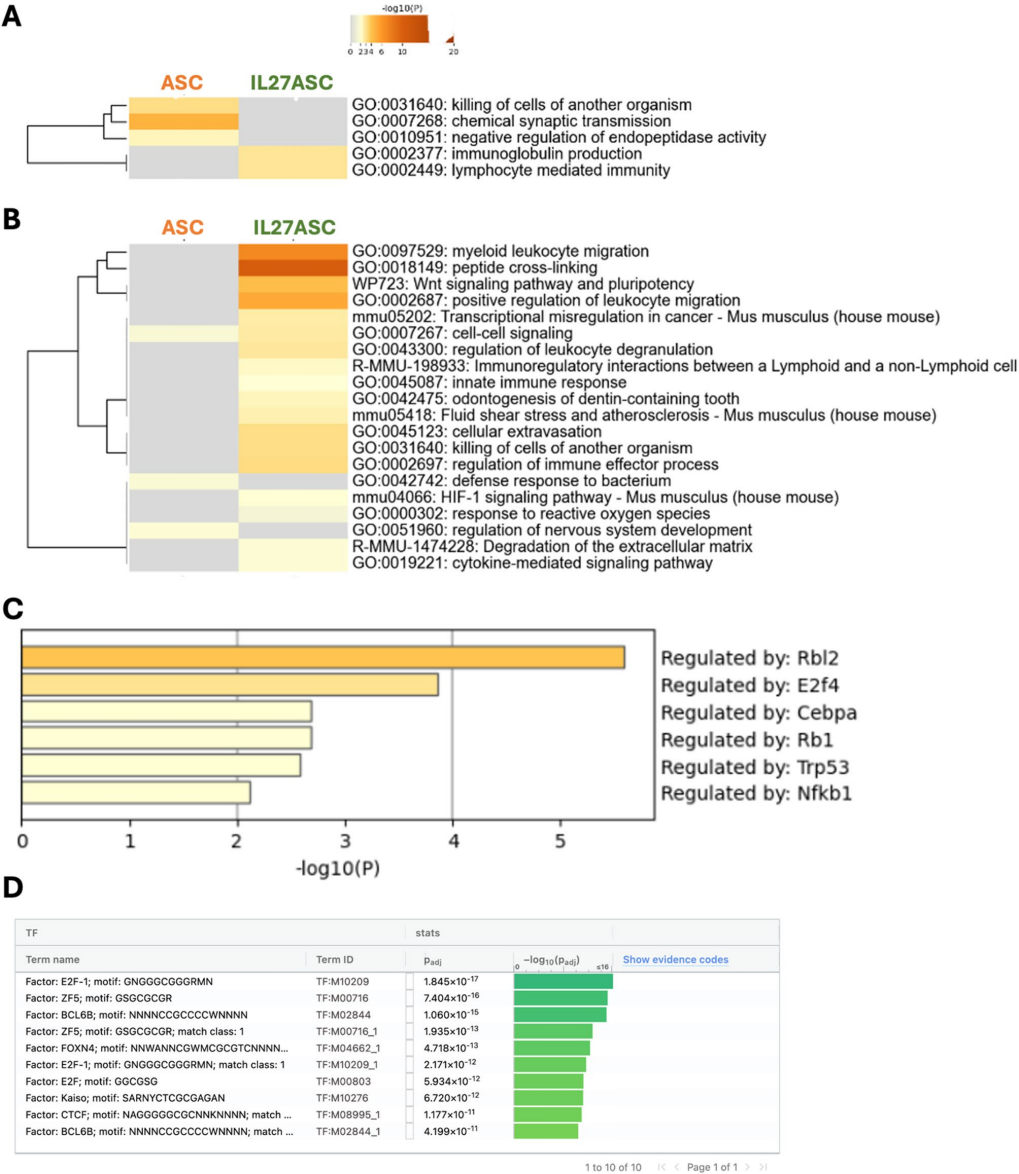
Pathway enrichment comparison ASC vs. IL-27 ASC groups relative to LPS. DEGs that were upregulated (**A**) and downregulated (**B**) in both ASC and IL-27 ASC groups relative to LPS were analyzed using Metascape, and the enriched pathways are displayed as heatmap. (**C**) Enriched transcription factors derived from downregulated DEGs in the IL-27 ASC group relative to the ASC group

Thus, to further understand the specific mechanisms and pathways involving the DEGs identified, we analyzed upregulated and downregulated DEGs using Metascape [29]. With this analysis, we determined the enriched pathways involved in the treatment of ASC and IL-27 ASC in the LPS-induced ARDS model (Fig. 8). The upregulated DEGs showed that ASC-enriched pathways were related to the *killing of cells of another organism*,* chemical synaptic transmission*, and *negative regulation of endopeptidase activity*, while IL-27 ASC enriched pathways related to *immunoglobulin production*, and *lymphocyte mediated immunity* (Fig. 8A). In contrast, notable downregulated DEGs in the IL-27 ASC group were related to processes that are involved in *myeloid leukocyte migration*,* regulation of leukocyte degranulation*,* cellular extravasation*,* innate immune response*,* regulation of immune effector process*,* cytokine-mediated signaling pathway*,* response to reactive oxygen species*, and *degradation of the extracellular matrix* (Fig. 8B). This suggests that IL-27 ASC treatment likely reduces pathways involved in neutrophil migration and degranulation, reducing inflammation through reactive oxygen species, in addition to reducing innate immunity while promoting adaptive immunity. The transcription factors *Rbl2*,* E2f4*,* Cebpa*,* Rb1*,* Trp53*, and *Nfkb1* were enriched in the downregulated DEGs in IL-27 ASC compared to ASC (Fig. 8C). Furthermore, we examined the upregulated transcripts from IL-27 ASC relative to ASC, utilizing the ~ 700 most upregulated transcripts in a *g: Profiler* analysis (via TRANSFAC). We identified E2F1 as a key regulator, suggesting IL-27 may influence cell cycle pathways (Fig. 8D). Among the top 300 transcripts, several transcripts (Grb2, Mx1, Hmgb1, and Nod2) were known to be IL-27 pathway targets, while others (Rbl2, Rps6ka4, and Cul4b) are linked to STAT1/3 and E2F1 signaling, suggesting activation of immune and proliferative responses. The presence of IRF3-associated genes also might support a role for IL-27 in antiviral defense. In combination, the findings presented in Fig. 8 suggest that IL-27 ASC treatment might dampen innate immune responses and promote adaptive immunity and cell cycle regulation.

**Fig. 9:**
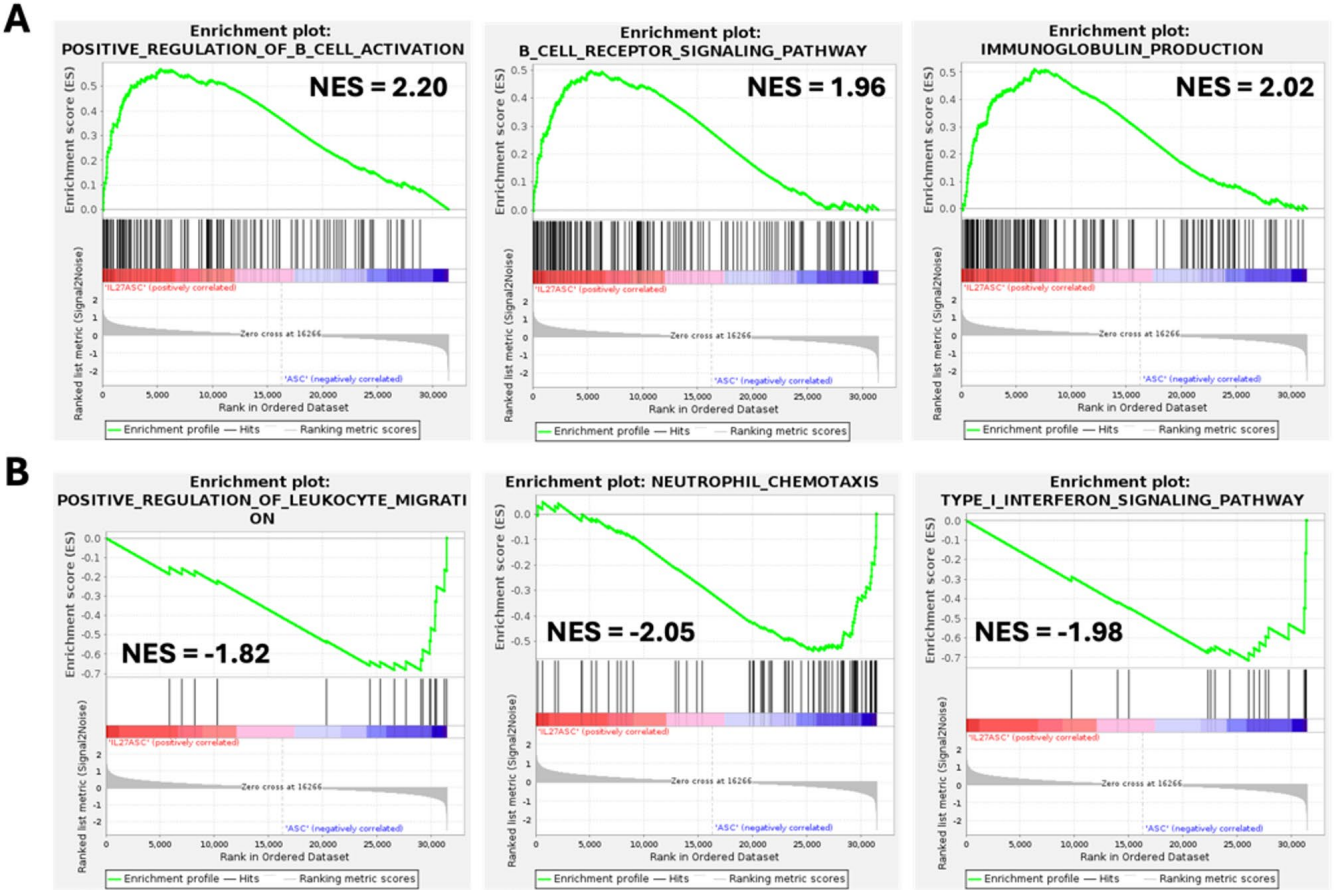
Enrichment plots from IL-27 ASC treatment of LPS-induced mouse lungs. Representative enrichment plots obtained from GSEA analysis of expressed genes ranked by fold change of expression. Gene sets for the biological processes that were positively **A** and negatively **B** enriched with IL-27 ASC treatment compared to ASC are shown

Gene Set Enrichment Analysis (GSEA) of the DEGs further identified gene sets that were enriched at a false discovery rate (FDR) < 25%. This threshold means a result is 75% likely not a false positive and is considered reasonable to identify hypotheses for further exploratory research. The main statistic used to compare the enrichment across gene sets is the normalized enrichment score (NES), which accounts for differences in gene set size. A positively enriched gene set would have an NES > 0, while negatively enriched gene sets would have an NES < 0, depending on whether the genes are up or downregulated, respectively. GSEA results showed that IL-27 ASC was positively associated with the regulation of *B cell activation*, *B cell receptor signaling pathway*, and *immunoglobulin production* when compared to the ASC group (Table 2; Fig. 9). In addition, IL-27 ASC was also negatively associated with *neutrophil chemotaxis*, *leukocyte migration*, *T cell migration*, and *T cell proliferation* relative to ASC. IL-27 ASC showed a negative association with signaling pathways such as *Type I interferon signaling pathways* and *positive regulation of Interleukin-6 production.* Overall, the key interferon and cytokine related pathways from a GSEA analysis could be summarized as centering on STAT-mediated pathways, in particular STAT1 (Suppl. Fig. S7).

**Table 2:** Immune-related GO pathways enriched in the IL-27-ASC group relative to the ASC group

Enriched GO pathways	NES	FDR q-val
Positive regulation of B cell activation	2.1956280	0
B cell receptor signaling pathway	1.9571126	0.013793026
Immunoglobulin production	2.0175025	0.006734313
Neutrophil chemotaxis	-2.0502600	0.00452886
Leukocyte migration	-1.8244572	0.034863032
Positive regulation of T cell migration	-1.9173888	0.018630398
T cell proliferation	-1.8291669	0.03378396
Type I Interferon signaling pathway	-1.9765921	0.011630286
Positive regulation of Interleukin-6 production	-1.9016380	0.021303076

### Estimation of immune infiltration in the lung tissues

Given the differences in enriched pathways and processes across the treatment groups, we sought to determine differences in immune cell composition within the lung tissues. DEGs derived from the RNA-seq data of LPS-induced mouse lung tissues were analyzed using TIMER 2.0 [30], which allows for an estimation of immune cell composition using data from bulk RNA-seq samples.

**Fig. 10:**
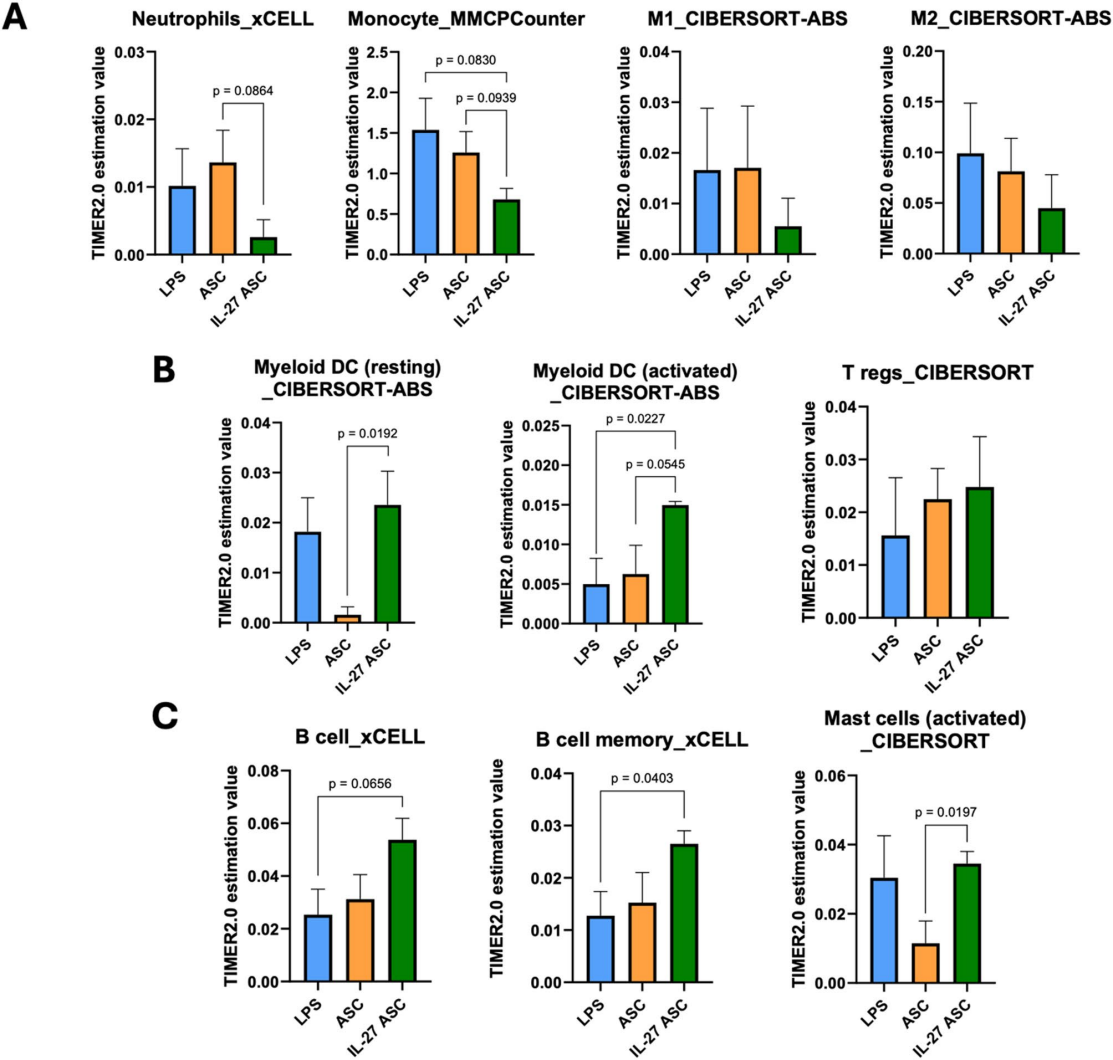
Estimation of immune cells infiltration composition in mouse lung tissues. Differentially expressed genes (DEGs) from RNA sequencing (RNA-seq) of mouse lung tissues (72 h post-LPS induction) were analyzed using TIMER 2.0. Unpaired t-test was performed using GraphPad Prism. Data are represented as mean abundance estimation value ± SEM (*n* = 4 samples per group)

Aligned with our observations in BALF and histological images, immune cell infiltration analysis estimated a trend of reduced neutrophil numbers in the IL-27 ASC group compared to the ASC group (Fig. 10A). A reduced number of monocytes/macrophages was also observed in the IL-27 ASC groups compared to the LPS and ASC groups. Notably, M1 macrophages, had the lowest estimated value in the IL-27 ASC group compared to either LPS or ASC alone. Interestingly, there was also a decrease in M2 macrophages in the IL-27 ASC group, though their numbers remained higher than M1 macrophages. This suggests that rather than the absolute number of M1 or M2 macrophages being crucial, the ratio or balance between M1 and M2 appears to be key in regulating inflammation, as suggested by previous studies [45, 46].

IL-27 ASC also seems to play a role in significantly driving the number and activation of myeloid dendritic cells (DCs), Tregs, B cells, and mast cells compared to the ASC group (Fig. 10B). This supports our pathway enrichment and serum analysis results, in which the upregulation of B cell number is consistent with the positive enrichment of immunoglobulin production, B cell activation, positive regulation of T cell migration and proliferation, and the upregulation of GM-CSF levels that typically correlate with the accumulation of DCs in inflamed lungs [47]. In the ARDS context, the adoptive transfer of Tregs in an LPS-induced ARDS in vivo model has been shown to promote lung injury resolution and suppression of TNF-α production in macrophages [48]. Similarly, higher levels of memory B cells have been associated with better prognosis in ARDS patients [49]. In contrast, there are not many studies on mast cells in the ARDS context but they are typically not present during the initial stages and are upregulated during the reparative or resolution stage of the inflammation [50]. Thus, taken together, the immune infiltration estimation data suggest that IL-27 ASC treatment reduces the number of neutrophils and macrophages (key players in the innate immunity), modulates the balance between M1/M2 macrophages, and promotes the adaptive immunity to trigger the repair of lung tissues.

## Discussion

Despite the advancement in many therapeutics across inflammatory conditions, ARDS patients still lack a definitive cure, relying on palliative care to ease their symptoms. This highlights the dire need for targeted therapeutic strategies to treat ARDS patients. In recent years, there has been a rise in studies using mesenchymal stromal cells (MSC) from various sources as a feasible therapeutic approach due to the innate anti-inflammatory, anti-apoptotic, and pro-angiogenic effects of MSC [51]. A clinical trial conducted by Matthay et al. demonstrated the safety and limited adverse events in using bone-marrow MSC to treat ARDS [12]. However, the study failed to show a significant difference in the efficacy of MSC treatment. Our use of adipose-derived MSC (ASC) as a gene carrier for IL-27 expression represents a novel therapeutic strategy aimed at combining the innate anti-inflammatory properties of ASC with the tissue repair-promoting effects of IL-27, as well as harnessing the ability of IL-27 to rebalance innate and adaptive immunity.

Collectively, our in vitro and in vivo results show promise in using IL-27 ASC as a therapeutic candidate to reduce inflammation in ARDS. Results from the in vitro studies showed a significant reduction in pro-inflammatory cytokines, including IL-8, IL-1β, IL-6, and TNF-α. Contrary to findings from other studies, our observations showed that ASC in an LPS-induced model showed a pro-inflammatory phenotypic profile. However, when ASC were transfected with an IL-27-expressing plasmid, pro-inflammatory cytokine production was suppressed, and this effect was maintained even at high concentrations of LPS. The immunosuppressive effect of IL-27 ASC was further supported by conditioned media (CM) experiments, where RT-qPCR analyses confirmed that IL-27 ASC treatment maintained or significantly reduced pro-inflammatory cytokine gene expression in lung epithelial cells and macrophages, both in monoculture and co-culture settings. While we did not perform multiplex cytokine profiling on conditioned media in this study, we recognize the importance of comparing the secretomes of ASC and IL-27 ASC. Future studies will include comprehensive analysis of cytokines, including IL-27, to better understand the contribution of ASC-derived factors. Notably, in another ongoing study, IL-27 ASC-conditioned media has been more effective at blocking viral entry into HEK293 cells than media from IL-27-expressing muscle cells, suggesting that the cellular context of IL-27 expression may influence its bioactivity.

The promising results from the in vitro studies provided us with sufficient evidence to explore an in vivo model. In our LPS-induced ARDS in vivo model, IL-27 ASC showed superior therapeutic outcomes, with lower lung injury scores, fewer infiltrating cells, decreased total protein concentrations, and reduced pro-inflammatory cytokine gene expression compared to other treatment groups. Although the ASC group exhibited some histological improvement, the lung injury score did not reach statistical significance. This discrepancy may be attributed to inter-sample variability and the influence of heavily weighted parameters in the scoring system, such as neutrophil presence in alveolar spaces. These findings aligned with the work of Jung et al., who observed that hASC alone did not attenuate an LPS-induced ARDS model [52]. As seen in the results of our in vitro studies, it is likely that the inflammatory microenvironment induced by LPS alters the phenotypic profile of ASC. A previous study by Rolandsson Enes et al. [53] showed that human MSC exposed to BALF from ARDS patients displayed upregulation of pro-inflammatory cytokines. Our results were further verified by RNA-seq data that demonstrated the addition of an IL-27-expressing plasmid to ASC reduced pathways and processes related to pro-inflammatory cytokine production, neutrophil migration and degranulation, while also rebalancing the innate and adaptive immunity responses – effects that were not achieved in the ASC group alone.

In addition to downregulating pro-inflammatory cytokines, IL-27 ASC slightly upregulated the gene expression of IL-10Rα, an anti-inflammatory cytokine receptor. While statistically significant changes in IL-10 protein or gene expression were not detected in the serum or RNA (data not shown), it is possible that the peak expression occurred at an earlier timepoint. Elevated levels of IL-10Rα are associated with SOCS3 activity and STAT3 phosphorylation [54], both of which are key regulators of IL-6 signaling and JAK/STAT pathways by suppressing their downstream signaling pathways. Meanwhile, IL-27 predominantly promotes activation of STAT1/3, suggesting fine-tuned regulation of STAT1/3 signaling through IL-27 and IL-10Rα may play a crucial role in modulating inflammation in ARDS. However, it should also be noted that the IL-10 receptor is a heterotetrametric complex, composed of two IL-10Rα and two IL-10Rβ subunits. Our study focused specifically on the gene expression of IL-10Rα, which is crucial for IL-10 ligand binding. Thus, the role of IL-10Rβ role in downstream signaling should not be dismissed and the complete effect of IL-27 ASC on the IL-10 signaling pathway could be further explored. Regardless, the in vivo results support IL-27 ASC’s role in regulating inflammation. IL-27 ASC reduces pro-inflammatory cytokine gene expression while promoting anti-inflammatory functions, suggesting its potential as a therapeutic candidate for ARDS.

The findings from our serum data analysis demonstrated the enhanced ability of IL-27 ASC to modulate the levels of pro-inflammatory cytokines/chemokines related to hyperinflammation and immune cell recruitment, while promoting factors that support lung repair and protective processes. Regarding immune cell recruitment, both ASC and IL-27 ASC treatment groups reduced eotaxin, LIX, and IL-5 levels. While the roles of eotaxin and LIX in the ARDS context are better defined as eosinophil chemoattractant and neutrophil recruiters, respectively, the role of IL-5 has yet to be fully understood in the context of ARDS. One study demonstrated that blocking a subunit of IL-5 reduced hyperinflammation in ARDS [55], whereas another demonstrated a protective role for IL-5 in a bleomycin-induced lung injury model, suggesting a dual role depending on timing and ARDS phase [56]. Additionally, other immune recruiting chemokines/cytokines such as G-CSF, KC, MCP-1, MIP-1β, RANTES, and IFN-γ were elevated following ASC treatment but reduced in the IL-27 ASC group, suggesting that this modulation is specific to IL-27 ASC. The observed increase in IL-6 and MCP-1 in the ASC group contrasts with reports of ASC-mediated suppression in ALI models. However, our heatmap reflected protein-level cytokine expression in serum at a single time point, capturing a snapshot rather than a longitudinal profile. ASC responses are known to be dynamic and context-dependent, particularly in inflammatory environments. Notably, IL-6 and MCP-1 levels were reduced in the IL-27 ASC group, suggesting IL-27 may modulate ASC inflammatory activity. This dual and time-sensitive nature of ASC cytokine modulation has also been observed clinically [57], supporting a broader anti-inflammatory potential for IL-27. Consistent with this, our group previously demonstrated in a collagen antibody-induced arthritis (CAIA) model that IL-27 gene delivery reduced key inflammatory cytokines including G-CSF, IFNγ, IL-1β, IL-13, IL-9, and TNF [55], mirroring the cytokine reductions observed in our lung injury model.

IL-27 ASC effects also impacted cytokines that were related to inflammatory mediators. The key signaling pathways that regulate the production of these cytokines/chemokines appear to be IFN-γ, IL-17, and IL-6 signaling, which can be modulated by IL-27. IL-27 inhibits IL-17 production in Th17 cells [58], independent of IL-10. Additionally, IL-27 acts as an antagonist of IL-6 signaling, suppressing IL-6 mediated Th17 production [6]. While IFN-γ, TNF-α, and IL-6 generally are classified as pro-inflammatory, their exact role in ARDS can be contradictory. Recent studies have shown that IFN-γ can have a protective role in ARDS, in both the exudative and fibrotic phases, yet only when its levels are tightly regulated within a critical time window, i.e., during the first 24 h after induction of cytokine release syndrome [59]. Similar to IFN-γ, IP-10 levels are typically upregulated during the early and progressive stages of ARDS but decrease as the disease reaches the convalescent stage [60]. At controlled levels, TNF promotes tissue repair and healing, but continuous elevated levels can lead to organ injury [61]. IL-6 is generally known as a pro-inflammatory cytokine, however, due to its dual role, whether the upregulation contributes to organ injury or prevents it remains controversial, as some studies suggest a protective role in ARDS [62]. Perhaps for IFN-γ, TNF, and IL-6 a tight regulation within a critical time window is necessary to harness their beneficial effects during inflammation. Collectively, this suggests that cytokines with a pleiotropic nature require tight regulation within a critical time window to generate a beneficial therapeutic effect, and our in vivo studies showed promising potential of IL-27 ASC in modulating the levels of these cytokines.

Additionally, we also observed the effects of IL-27 ASC in promoting tissue repair in our LPS-induced ARDS model by decreasing IL-28B and GM-CSF levels in the serum. IL-28B (IFN-λ3) is a type III interferon that correlates with morbidity of SARS-CoV-2 associated ARDS and inhibits the proliferation and repair of lung epithelia [63]. Elevated IL-28B levels are also associated with pulmonary fibrosis in human serum and bleomycin-induced ARDS models [64]. Meanwhile, GM-CSF is an immune modulating cytokine that drives the differentiation of alveolar macrophages while also playing a role in maintaining the integrity of lung epithelium, helping to restore balance in the pulmonary immune system [65]. In ARDS patients, elevated GM-CSF levels in BALF were associated with antiapoptotic effects and improved epithelial barrier integrity and survival [47]. Thus, IL-27 ASC was able to modulate inflammation by suppressing fibrotic-promoting cytokines while inducing mediators that promote lung tissue repair.

Our data also indicated shifts in estimated immune cell populations following a IL-27 ASC treatment, including a reduction in neutrophils, a rebalancing of macrophages, and an increase in myeloid cells, B cells, Tregs, and mast cells. As the LPS-induced ARDS model reflects the acute inflammatory phase, we expected changes in neutrophils and macrophages, being the main players during hyper immunoactivation. Indeed, a trend of reduced estimated number of neutrophils was detected by TIMER2.0 analysis. Despite the changes in gene expression that showed a reduced pro-inflammatory profile, reductions were also estimated for both M1 and M2 macrophages. This leads us to believe that rather than the absolute number of each, the ratio or balance between M1/M2 could be instead crucial in regulating inflammation. IL-27 has been shown to influence innate immunity and macrophage polarization, including inhibition of the Nrf2/HO1 signaling pathway in sepsis-induced ARDS models, promoting M1 polarization and inhibiting M2 polarization [65]. While this may represent one mechanism, further investigation would be required to fully understand how IL-27 expressing ASC modulate macrophage dynamics. Previous studies have reported conflicting roles of IL-27 in sepsis and lung injury models. For example, Cao et al. [66] showed that IL-27 neutralization improved survival and bacterial clearance in a CLP-induced sepsis model, while Xiong et al. [67] found that recombinant IL-27 promoted M1 polarization and aggravated lung injury. These discrepancies may be attributed to differences in timing, delivery method, and disease model. Notably, both studies administered recombinant IL-27 prior to disease induction, whereas our study used ASC-mediated IL-27 delivery after LPS injury. This distinction may explain the contrasting outcomes and supports the context-dependent nature of IL-27’s immunomodulatory effects.

In our study, to further explore the molecular mechanisms underlying potential immunomodulatory effects, we analyzed differentially expressed genes and transcripts using Metascape and g: Profiler, respectively. The enrichment analyses suggested that ASC treatment upregulated pathways related to microbial killing and synaptic signaling, while IL-27 ASC treatment enhanced pathways associated with immunoglobulin production and lymphocyte-mediated immunity. Conversely, IL-27 ASC downregulated a gene profile involved in neutrophil migration, degranulation, and innate immune responses, suggesting a shift toward adaptive immunity. Transcription factor analysis identified regulators such as E2f1, Stat1/3, and Irf3, implicating IL-27 in immune modulation and cell cycle regulation. These findings support the hypothesis that IL-27 ASC treatment may act to dampen hyper-inflammation while promoting immune resolution and tissue repair. Recent work by Afsahi and Branson [68] further supports IL-27’s anti-inflammatory potential, demonstrating that T cells engineered to express IL-27 maintained effector function while reducing pro-inflammatory cytokine profiles in inflammatory environments.

Additionally, while not central to the initial phase of ARDS, the estimated trend in the increase of Tregs, B cells, DCs, and mast cells levels following IL-27 ASC treatment was also of interest to us since our samples were collected 72 h post-LPS induction, a closer time point towards the start of the ARDS resolution phase. Previous evidence supports a role for IL-27 in promoting Treg expansion, with a greater effect than IFN-γ and IL-10, through the activation of STAT1/3 [69]. Additionally, the ability of IL-27 to promote proliferation and activation of B cells has been described previously [70]. IL-27 has also been shown to act on DCs to promote the modulation of T cell responses [71] and the development of Tregs [72]. The effects of IL-27 on mast cells are not extensively studied in ARDS but have typically been explored in the context of asthma or allergy. IL-27 has been shown to inhibit mast cell activation and degranulation in vitro, but the complete abolishment of IL-27 signaling promotes hyperinflammation [73], suggesting that a very tight balance of IL-27 levels is required in modulating inflammatory responses. While there are some studies that describe contradictory findings, it should be noted that due to the pleiotropic nature of IL-27, its effects are typically context-dependent, varying with the disease context, dose, and timing of administration. Interestingly, in our study, IL-27 ASC gene therapy reduces inflammation in vitro and in an LPS-induced ARDS mouse model. This therapeutic approach likely modulates IL-27 activity differently when expressed by MSC, reducing the excessive inflammatory response and tissue damage characteristic of ARDS. However, further exploration is needed to clarify the exact mechanisms by which IL-27 activity is modulated when expressed by MSC and how immune cells are regulated by IL-27 ASC in the context of ARDS.

There are several limitations to our study. To date, there is no single mouse model that can fully replicate the clinical symptoms of human ARDS patients. In this work, we utilized an LPS-induced model which mimics common symptoms seen in ARDS, particularly during the exudative phase. Therefore, the conclusions drawn from this study are specifically limited to bacterially induced ARDS, as modeled by LPS exposure and should not be generalized to other ARDS etiologies. We recognize the variability of ARDS symptoms and etiology of ARDS, which stresses the importance of validating the therapeutic efficacy of IL-27 ASC in additional in vivo models to enhance clinical relevance. One example would be the bleomycin-induced lung injury model, to explore the therapeutic efficacy in lung fibrosis. This is an interesting model to evaluate IL-27’s capability in promoting tissue repair in addition to its anti-inflammatory properties. Additionally, the bleomycin-induced lung injury model is typically studied over the course of at least 7 days, thus allowing for a model that fits a longer study period. This could be beneficial for investigating the lasting effects of IL-27 ASC, as well as the persistence of ASC at the inflammatory site within the ARDS context. To further clarify the therapeutic potential and clinical relevance of IL-27 ASC, future studies should also incorporate additional models such as cecal ligation and puncture and intratracheal infection with pathogens. These models would allow for evaluation of IL-27 ASC in polymicrobial sepsis and pathogen-driven lung injury, thereby expanding the translational scope of our findings. Additionally, by taking advantage of ASC’s innate homing ability, we can also evaluate the effectiveness of IL-27 ASC as a targeted gene delivery system by comparing efficacy of other therapeutic administration routes. A further limitation was the relatively small sample size used in some pilot experiments, which may affect the generalizability of those specific findings. Future studies will include larger cohorts to enhance statistical power and reproducibility.

Another limitation of our ARDS in vivo study stemmed from the use of xenogeneic ASC as our gene carrier. Despite the inter-species limitations, such as differences in IL-27 receptor compatibility, human ASC have demonstrated effective immunomodulatory properties in murine models and reduced immune rejection risk due to their low MHC class II molecule expression [74]. Additionally, both human and mouse ASC have been shown to be efficient in attenuating LPS-induced murine models of lung injury, demonstrating that ASC can function across species barriers [50, 74–76]. While we demonstrated distinct profiles of differentially expressed genes, cell infiltrates, cytokines/chemokines in mouse serum, and histological evidence depending on the treatment groups, not all changes were statistically significant. We speculate that the collective action of other metabolites and secreted factors produced by the ASC, aside from IL-27, could also contribute to their therapeutic effects. Thus, perhaps utilizing an allogeneic ASC for our in vivo experiments could have enhanced the therapeutic effect of IL-27-expressing-ASC compared to xenogeneic ASC. We acknowledge that our studies also could be improved by including positive controls, comparison with other types of MSC, and expansion to more pathway-, dose-, and time-dependent groups, as well as comparing the effects of IL-27 alone with IL-27 expressing ASC. In addition, comparison studies between allogeneic and autologous ASC would be informative in future studies. While autologous ASC minimize the risk of immune rejection and graft-versus-host disease, they are less cost effective. Thus, allogeneic ASC offer a scalable, off-the-shelf alternative solutions that are suitable for broad patient populations. Comparing the efficacy of these strategies would help address the current limitations and enhance the translational potential of ASC therapies.

While ASC-based gene therapy may offer localized and sustained cytokine delivery, its clinical translation may be limited by cost and complexity. In contrast, recombinant IL-27 might allow for controlled dosing and scalability. However, our prior work [18–20] showed that gene-delivered IL-27 is comparably bioactive, and unpublished data suggest ASC-derived IL-27 may be more effective than other cell sources. The present study opens up new directions and opportunities, but these should be balanced by the need for further investigation to compare delivery platforms that can optimize IL-27 release prior to any clinical use.

## Conclusion

Our investigation into the therapeutic potential of IL-27 expressing stromal cell-based gene therapy shows promise in reducing inflammation while promoting tissue repair in the context of ARDS. The in vitro data demonstrated the effects of IL-27 ASC in reducing pro-inflammatory cytokine production in both mono- and co-culture settings involving lung epithelial cells and macrophages. Even in the presence of an inflammatory insult (LPS), the IL-27 ASC treatment group still suppressed pro-inflammatory production, as shown in vitro. This effect was further confirmed and amplified amplified in the LPS-induced in vivo model, where IL-27 ASC modulated chemokines/cytokines responsible for immune cell recruitment, rebalancing innate and adaptive immunity, and promoted the production of cytokines and activation of cells involved in tissue repair. Despite these findings providing a strong foundation for future research, more work is still needed to address the remaining limitations and bridge the gap to clinical translation.

## Supplementary Information

Below is the link to the electronic supplementary material.

Supplementary Material 1

## Data Availability

The RNAseq data is available at NCBI GEO datasets with the accession number GSE292564; https://www.ncbi.nlm.nih.gov/geo/query/acc.cgi?acc=GSE62564.

## Abbreviations

Anti-Anti: Antibiotic-antimycotic
APC: Antigen presenting cell
ARDS: Acute respiratory distress syndrome
ASC: Adipose derived mesenchymal stromal cells
ATCC: American type culture collection
BALF: Bronchoalveolar lavage fluid
Bregs: B regulatory cells
BEGM: Bronchial epithelial cell growth media
CCL: Chemokine ligand
CD: Cluster of differentiation
CM: Conditioned media
COVID-19: Coronavirus disease of 2019
CXCL: C-X-C motif chemokine ligand
DC: Dendritic cell
DMEM: Dulbecco’s modified Eagle’s Medium
EBI3: Epstein-Barr virus induced gene 3
FBS: Fetal bovine serum
FOXP3: Forkhead box P3
Gp130: Glycoprotein 130
hASC: Human adipose derived mesenchymal stromal cells
IFN: Interferon
IL: Interleukin
JAK: Janus kinase
LPS: Lipopolysaccharide
LUNG-SAFE: Large observational study to understand the global impact of severe acute respiratory failure
M1: Pro-inflammatory macrophage
M2: Anti-inflammatory macrophage
MCP: Monocyte chemotactic protein
MIP: Macrophage inflammatory protein
MSC: Mesenchymal stromal cells
NFκB: Nuclear factor kappa-B
NK: Natural killer
ORF: Open reading frame
RNA-seq: RNA sequencing
RPMI: Roswell Park Memorial Institute
SARS-CoV-2: Severe acute respiratory syndrome related coronavirus 2
STAT: Signal transducer and activator of transcription
TGF-β: Transforming growth factor beta
Th1: T helper type 1
Th17: T helper type 17
Th2: T helper type 2
TNF: Tumor necrosis factor
Tregs: T regulatory cells
