# Supplementary material for: Interleukin-27-adipose-derived mesenchymal stromal cell-based gene therapy attenuates inflammation in lipopolysaccharide-induced acute respiratory distress syndrome
Source: Stem Cell Res Ther. 2025 Sep 29;16:535. doi: 10.1186/s13287-025-04647-1 (PMC12481768; doi:10.1186/s13287-025-04647-1)
Supplement: Supplementary file 1 — Supplementary Material 1 [file 13287_2025_4647_MOESM1_ESM.docx]

# **Supplementary Materials**


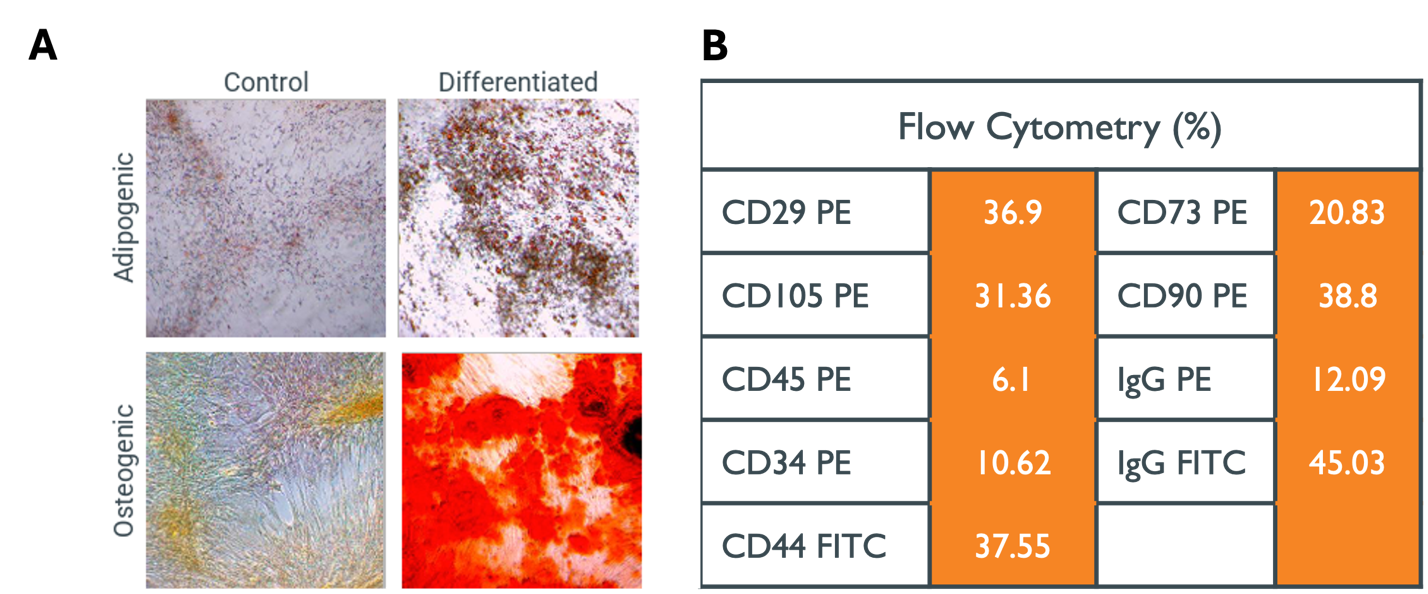


**Supplementary Figure S1**. **Human Adipose Mesenchymal Stromal Cells (ASC) Characterization performed by Obatala Sciences.** (A) Brightfield microscopy images at 10X magnification of adipogenic and osteogenic differentiation of ASC stained with Oil Red for lipid and Alizarin Red, respectively. (B) Summarized results from flow cytometry of ASC at passage 0 to detect canonical MSC (CD29, CD73, CD90, CD105, and CD44) and hematopoietic markers (CD45 and CD34).

**Supplemental Figure S2. Transfected human ASC. A)** Human ASC was transfected/co-transfected with either 10% of GFP-expressing plasmid, empty vector and 10% GFP-expressing plasmid, or IL-27 expressing plasmid and 10% GFP-expressing plasmid. Images were taken at either 24- or 48-h post transfection. B) Expression of IL27 subunits (IL27p28 and EBI3) detected following transfection with empty vector or IL27-encoding plasmid. Data represent mean fold change ± SD (n = 3) as assayed by quantitative RT-PCR; Welch’s t-test compares IL27 plasmid to respective controls


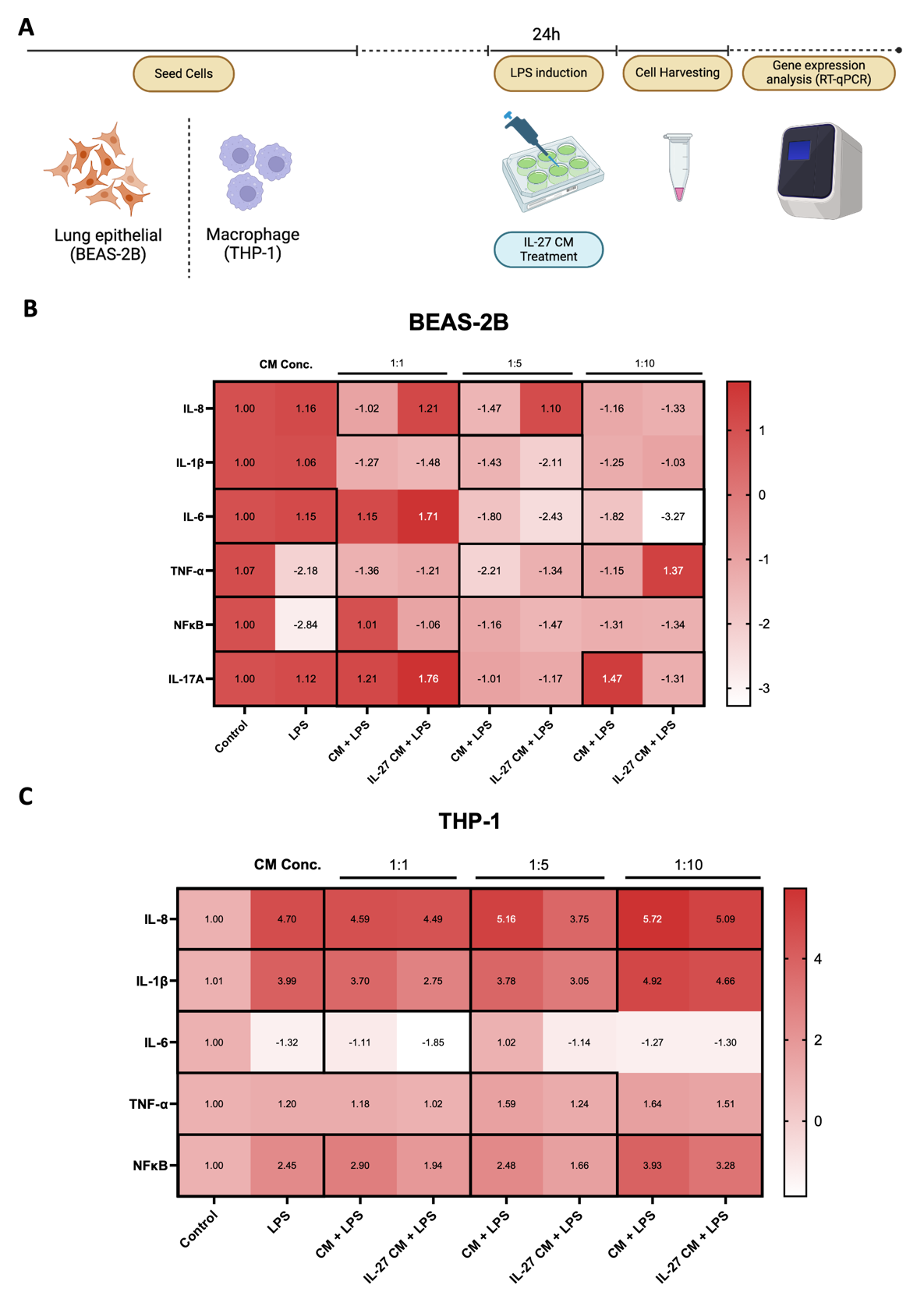


**Supplemental Figure S3. Effects of IL-27 hASC CM on LPS-induced lung epithelia and macrophage monoculture.** (A) Methodology overview of the monoculture LPS-induced ARDS model treated with and without IL-27 hASC CM at varying concentrations. Lung epithelia cell line (BEAS-2B) or macrophages (PMA-differentiated THP-1) were simultaneously treated with or without LPS (1000 ng/mL) and IL-27 hASC CM, as indicated. Gene expression heatmap of BEAS-2B (B) and THP-1 (C) cells treated with or without LPS and/or IL-27 hASC CM. Gene expression fold changes were normalized to the endogenous control (GAPDH). Upregulated genes are represented in red and downregulated in white. One-way ANOVA were conducted using GraphPad Prism and data are presented as the mean. Black borders around two groups denote statistical significance of at least p < 0.05.


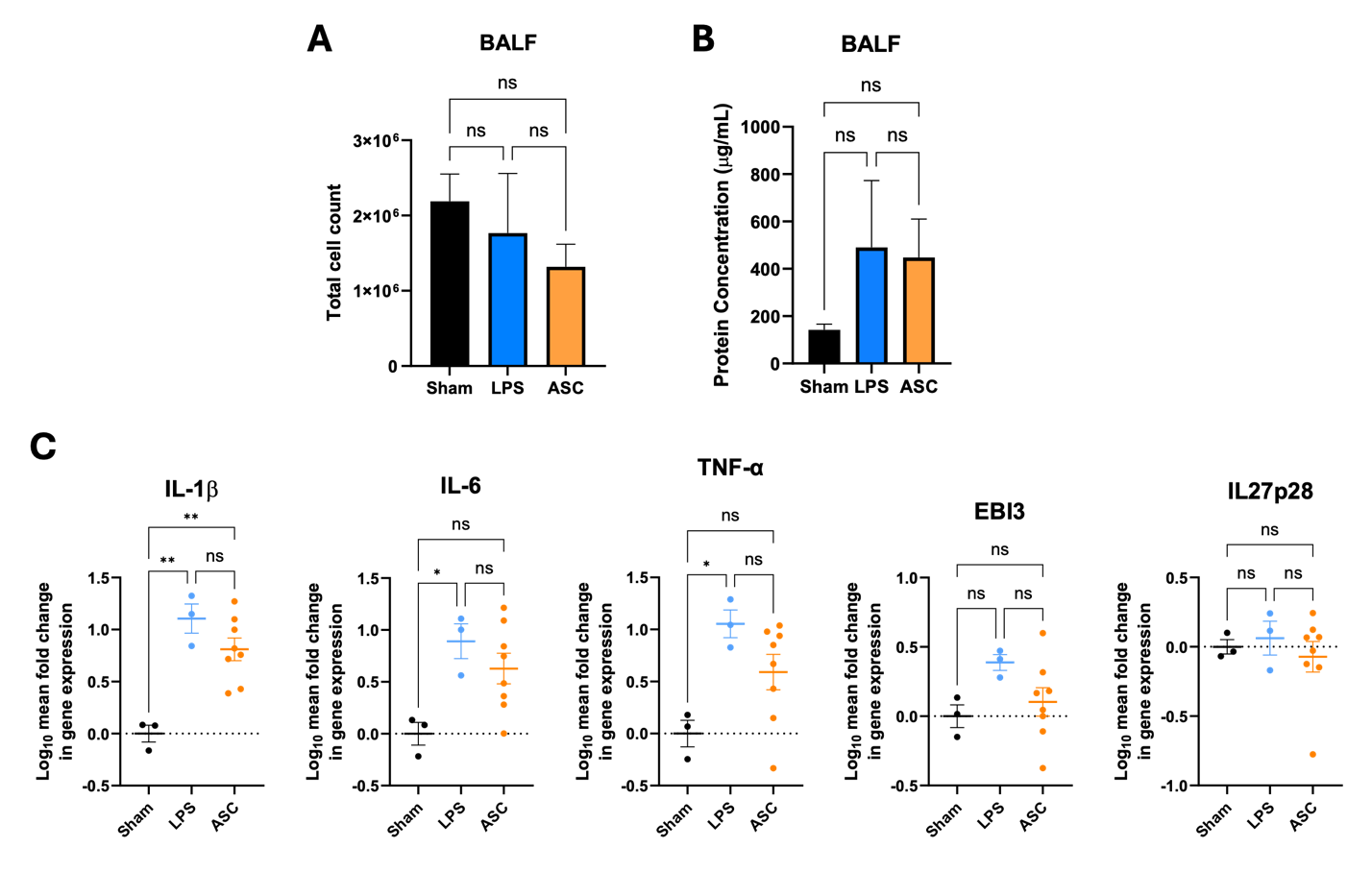


**Supplemental Figure S4. Effects of hASC administration alone on non-invasive LPS-induced *in vivo* ARDS model.** Male C57BL/6 mice (n = 3-8 per group) were subjected to non-invasive intratracheal injection of PBS or LPS (5 mg/kg). 24h post LPS-induction, mice were administered PBS or ASC (5 x 10^5^ cells / mouse) through a non-invasive intratracheal injection. Mice were sacrificed at 72h post LPS induction. (A) Total cell count and (B) total protein concentration of BALF at 72 h. (C) Gene expression fold change of pro-inflammatory cytokines (IL-1β, IL-6, TNF-α) and IL-27 subunits (EBI3 and IL27p28) at 72h. One way ANOVA with Tukey’s multiple comparisons test was performed using GraphPad Prism. Data are presented as mean ± SEM. *p < 0.05, **p < 0.01, ***p < 0.001 ****p < 0.0001.


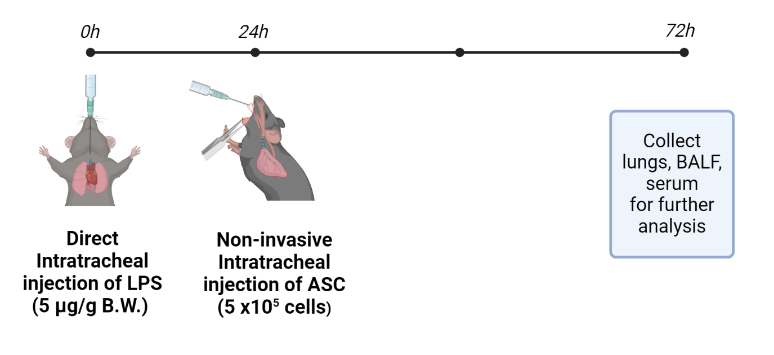


**A**

**B**

**C**

**Supplemental Figure S5. Effects of IL-27 expressing hASC administration on a direct intratracheal LPS induction of *in vivo* ARDS model.** (A) Methodology overview. Male C57BL/6 mice (n = 4-5 per group) were subjected to direct intratracheal injection of LPS (5 mg/kg). 24h post LPS-induction, mice were administered with PBS, ASC (5 x 10^5^ cells / mouse) or IL-27 ASC (IL-27 expressing hASC) through a non-invasive intratracheal injection before mice were sacrificed at 72h post LPS induction. (B) Total cell count of BALF at 72h. (C) Gene expression fold change of pro-inflammatory cytokines (IL-1b, IL-6, TNF-a) at 72h. One way ANOVA with Tukey’s multiple comparisons test was performed using GraphPad Prism. Data is represented as mean ± SEM. *p ≤ 0.05, **p ≤ 0.01, ***p ≤ 0.001 ****p < 0.0001.


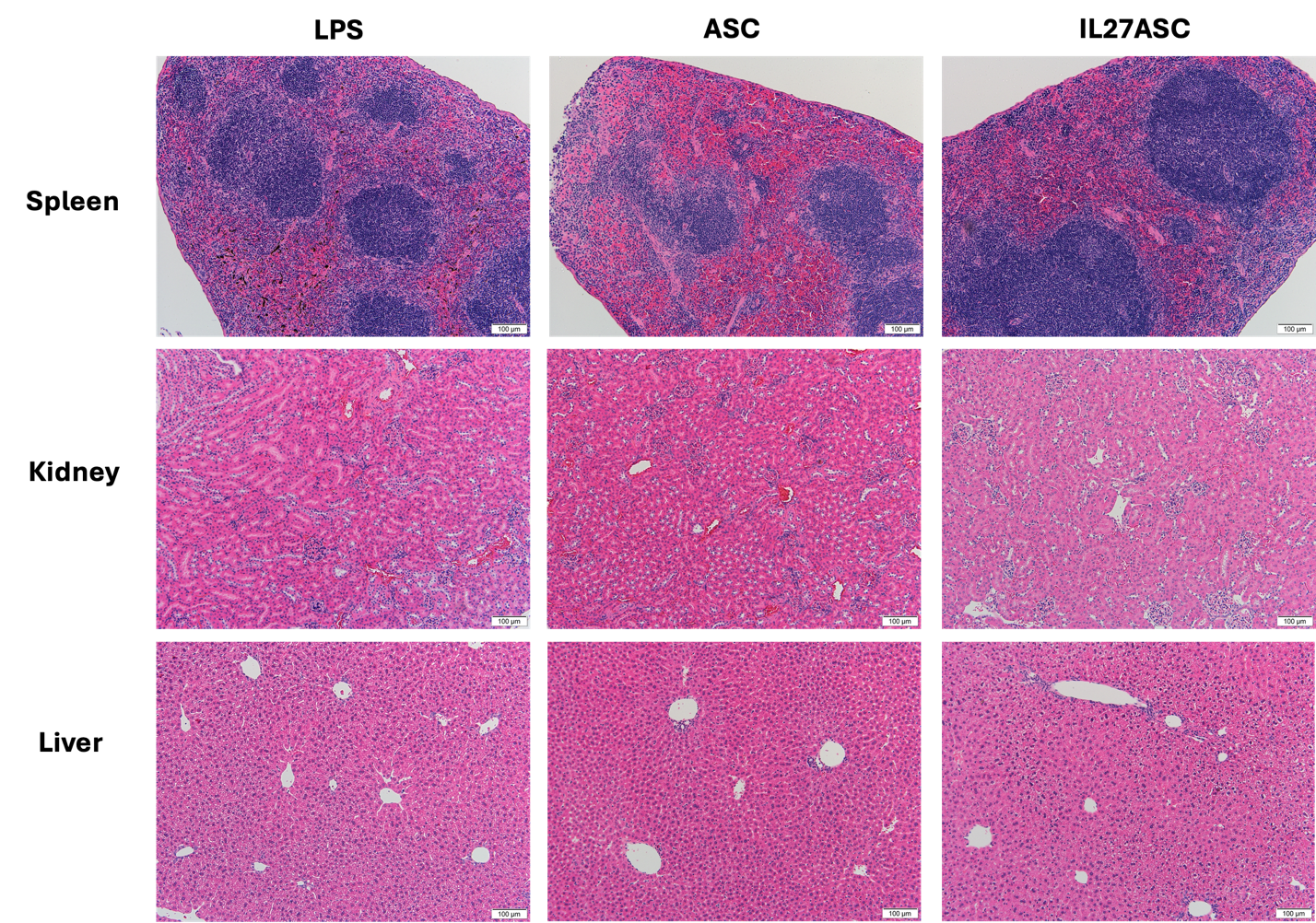


**Supplemental Figure S6. Representative histological images (H&E) of spleen, kidney, and liver from LPS-induced *in vivo* ARDS model.** Images were taken at 72 h post LPS induction using Olympus BX 53 at 10X magnification.

**Suppl. Fig. 7.** GSEA reveals key immune processes altered by IL27 treatment, highlighting STAT1 as a central node, particularly in interferon and cytokine signaling pathways.
